# The Relevance of the SH2 Domain for c-Src Functionality in Triple-Negative Breast Cancer Cells

**DOI:** 10.3390/cancers13030462

**Published:** 2021-01-26

**Authors:** Víctor Mayoral-Varo, María Pilar Sánchez-Bailón, Annarica Calcabrini, Marta García-Hernández, Valerio Frezza, María Elena Martín, Víctor M. González, Jorge Martín-Pérez

**Affiliations:** 1 Instituto de Investigaciones Biomédicas A, Sols/Dpto. Bioquímica (CSIC/UAM), Arturo Duperier 4, 28029 Madrid, Spain; vmayoral@iib.uam.es (V.M.-V.); Pilar.Sanchez@mdc-berlin.de (M.P.S.-B.); annarica.calcabrini@iss.it (A.C.); 2 Max Delbrück Center for Molecular Medicine (MDC), Robert-Rössle-Str. 10, 13092 Berlin, Germany; 3 National Center for Drug Research and Evaluation, Istituto Superiore di Sanità, Viale Regina Elena 299, 00161 Rome, Italy; 4 Grupo de Aptámeros, Servicio Bioquímica-Investigación, IRYCIS-Hospital Ramón y Cajal. Ctra. Colmenar Viejo km 9100, 28034 Madrid, Spain; marta.garcia@hrc.es (M.G.-H.); valerio.frezza@hrc.es (V.F.); m.elena.martin@hrc.es (M.E.M.); victor.m.gonzalez@hrc.es (V.M.G.); 5 Instituto de Investigaciones Sanitarias del Hospital La Paz (IdiPAZ), Paseo de la Castellana 261, 28046 Madrid, Spain

**Keywords:** triple-negative breast cancer (TNBC), c-Src, SH2 domain, inactivating point mutation, aptamers

## Abstract

**Simple Summary:**

Triple-Negative breast cancers (TNBC) have not specific therapeutic targets and are considered the most aggressive mammary tumors. c-Src controls several cellular processes: proliferation, differentiation, survival, motility, and angiogenesis. Alteration of c-Src functionality, by increasing its expression and/or its kinase activity, is associated to progression and metastasis of tumors in mammary gland, pancreas, colon, brain, and lung. However, c-Src tyrosine kinase inhibitors alone are not fully clinically effective, suggesting that c-Src adapter SH2/SH3 domains may be important. We questioned whether the SH2-c-Src domain is relevant for tumorigenicity of TNBC SUM159 and MDA-MB-231 human cell lines. Conditional expression of SH2 and SH3 inactivating mutants in these TNBC cells, or transfection of aptamers directed to SH2, allowed us to show that this domain is required for their tumorigenesis. Therefore, the SH2-c-Src domain could be a promising therapeutic target that, combined with c-Src kinase inhibitors, may represent a novel therapeutic strategy for TNBC patients.

**Abstract:**

The role of Src family kinases (SFKs) in human tumors has been always associated with tyrosine kinase activity and much less attention has been given to the SH2 and SH3 adapter domains. Here, we studied the role of the c-Src-SH2 domain in triple-negative breast cancer (TNBC). To this end, SUM159PT and MDA-MB-231 human cell lines were employed as model systems. These cells conditionally expressed, under tetracycline control (Tet-On system), a c-Src variant with point-inactivating mutation of the SH2 adapter domain (R175L). The expression of this mutant reduced the self-renewal capability of the enriched population of breast cancer stem cells (BCSCs), demonstrating the importance of the SH2 adapter domain of c-Src in the mammary gland carcinogenesis. In addition, the analysis of anchorage-independent growth, proliferation, migration, and invasiveness, all processes associated with tumorigenesis, showed that the SH2 domain of c-Src plays a very relevant role in their regulation. Furthermore, the transfection of two different aptamers directed to SH2-c-Src in both SUM159PT and MDA-MB-231 cells induced inhibition of their proliferation, migration, and invasiveness, strengthening the hypothesis that this domain is highly involved in TNBC tumorigenesis. Therefore, the SH2 domain of c-Src could be a promising therapeutic target and combined treatments with inhibitors of c-Src kinase enzymatic activity may represent a new therapeutic strategy for patients with TNBC, whose prognosis is currently very negative.

## 1. Introduction

The Src family of non-receptor tyrosine kinases (SFKs) is composed of nine members, and it has a modular structure, containing the SH2 and SH3 (Src homology domains 2 and 3), which are involved in protein-protein interactions with tyrosine phosphorylated proteins or with proteins containing proline rich sequences, respectively [1,2]. These two domains are also present in many other adapter and regulatory proteins, and facilitate the formation of intracellular signaling complexes [3]. While the activity of c-Src, the prototype of the SFKs, is mainly modulated by phosphorylation, the SH2 and SH3 domains are also required for the conformational changes associated with its cellular distribution, kinase activity, and cell functionality [4,5,6,7]. c-Src plays a key regulatory role of many cellular processes, including proliferation, differentiation, survival, motility, and angiogenesis. Therefore, alteration of c-Src functionality, by increasing its expression and/or its kinase activity, has been associated to progression and metastasis of tumors in the mammary gland, pancreas, colon, and lung [1,5,6,7,8].

Breast tumors are diverse, and they have been classified according to their genetic and histological characteristics [9,10]. Among them, the basal triple negative breast cancer (TNBC) does not express estrogen and progesterone receptors (ER^−^, PR^−^), nor overexpresses HER2, and usually has an inactive p53 mutant [10,11,12]. TNBCs do not have specific therapeutic targets and are considered the most aggressive mammary tumors, with a tendency to metastasize mainly into the lung, brain, and bone [13,14].

Experimentally, inhibitors of c-Src kinase activity, or its suppression, block proliferation, survival, migration, and invasion, as well as tumorigenesis in vivo [15,16,17]. However, inhibitors of c-Src tyrosine kinase activity alone do not appear to be fully clinically effective [8,18], suggesting that its adapter domains may play an important role for c-Src functionality in tumorigenesis. In this context, expression of c-Src with point-inactivating mutations at either SH2 or SH3 domains, which conferred stimulation of its kinase activity, blocks the prolactin-induce activation of Jak2 in MCF7 [19]. In mouse SYF fibroblasts, expression of c-Src-R175*L* prevents Fak auto-phosphorylation (pY397), malignant transformation, motility defects, and focal adhesion formation, indicating the relevance of the SH2 domain of c-Src [20]. The SH2 domain of c-Src interacts with the pY397-Fak facilitating the open conformation of c-Src that activates its kinase activity and, in turn, protects pY397-Fak from phosphatases [21,22]. In addition, c-Src phosphorylates Fak on several tyrosine residues, thus promoting cellular signaling and tumor progression [6,23,24].

Small molecules, such as inhibitory peptides and non-peptides, have been used to block the SH3/SH2 domains of c-Src [25,26,27,28,29] with a relative success. Aptamers are single stranded oligonucleotides (DNA or RNA) that bind to proteins with high affinity and specificity, blocking their functionality. They have been used for diagnosis and therapy in several infectious, inflammation, vascular diseases, as well as in other pathologies including breast cancer [30,31,32]. 

Here, we analyzed the role of the adapter domain of c-Src in the in vitro tumorigenic properties of SUM159PT (from now on SUM159) and MDA-MB-231 TNBC cell lines. We found that the conditional expression of c-Src variants with suppression of SH2 functionality caused profound effects on the behavior of these triple negative cell lines. Consistently, two different aptamers directed to SH2-c-Src inhibited proliferation, migration, and invasiveness of both SUM159 and MDA-MB-231 cells. Thus, the SH2-c-Src domain appears to play a crucial role in TNBC tumorigenesis.

## 2. Results

### 2.1. c-Src Variants of the SH2 Adapter Domain

In the studies presented here we used two different triple negative breast cancer (TNBC) cell lines, SUM159 and MDA-MB-231. Although SUM159 and MDA-MB-231 are both Basal-Mesenchymal TNBC cell lines with a spindle phenotype, they show differences in deleted and mutated genes. Furthermore, previously published data from the laboratory using both SUM159 and MDA-MB-231 cells showed that they differ in some signaling responses [16]. All together, we can conclude that even if both are representing TNBC cells, their cellular behavior could diverge.

**Figure 1:**
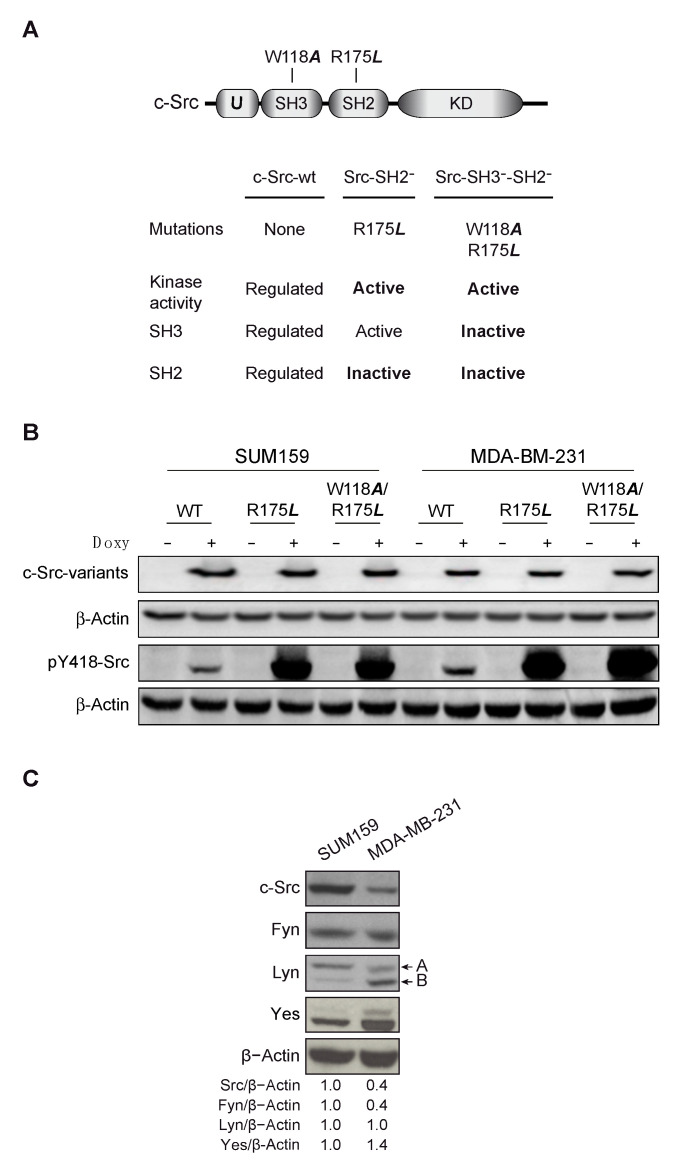
c-Src variants and expression of Src kinases in SUM159 and MDA-MB-231 cells. (**A**) Schematic design of c-Src and the variants employed in this study, which were conditionally expressed (Tet-On system) upon addition of doxycycline (Doxy, 0.2 μg/mL) to the cell culture. The R175***L*** mutation inhibits both intra- and inter-molecular interactions of the SH2 domain of c-Src. The W118***A***/R175***L*** double mutation inhibits both the SH2 and SH3 domains. (**B**) Induction of chicken c-Src variants by Doxy was detected by Western blot (WB) using the EC10 mouse monoclonal antibody that specifically recognizes chicken c-Src and β-Actin as a loading control. (**C**) Comparative expression of Src kinases in SUM159 versus MDA-MB-231 cells determined by WB. Actin was used as a loading control and the ratio of kinase/actin in SUM159 was considered as 1.

To analyze the role of the SH2 adapter-domain of c-Src in the in vitro tumorigenic properties of SUM159 and MDA-MB-231 cell lines, we conditionally expressed (Tet-On system) chicken c-Src variants with point mutations inactivating this domain (Figure 1A). It should be pointed out that chicken c-Src could replace human c-Src functionality [16], as they have more than 94% identity at the amino acid sequence [33]. Nevertheless, the EC10 mouse monoclonal antibody (Millipore, no. 05-185) specifically recognizes chicken c-Src, making it possible to determine by Western blot (WB) the expression of c-Src variants in the presence of the endogenous human c-Src of SUM159 and MDA-MB-231 cells.

We used the R175***L*** point mutation (SH2) affecting intra- and inter-molecular c-Src interactions, preventing c-Src from being in the close configuration, consequently, c-Src-R175*L* has a constitutive tyrosine-kinase activity [34]. The c-Src-SH2-SH3 double variant (c-Src-W118*A*-R175*L*) [19], which also has a constitutive tyrosine-kinase activity, was also employed to test for the impact of the SH3 domain in SH2 functionality. To avoid undesirable effects due to the insertion of the c-Src variants in the genome of the cells upon transfection, each cell line was made of a pool of, at least, three positive independent clones. Since we are studying the role of SH2 domain in TNBCs SUM159 and MDA-MB-231, we expressed the wild type c-Src (c-Src-wt) to compare the results obtained with the SH2 mutant. As observed in Figure 1B, the chicken c-Src variants were induced by the addition of Doxy to culture media (0.2 μg/mL, 72 h) at similar levels in SUM159 and MDA-MB-231. Analyses of the degree of activation of c-Src chicken variants by determining the autophosphorylation at Y418 showed that the expression of c-Src-wt slightly increased the levels of phosphorylation at Y418 in both cell lines. In contrast and consistent with the scheme presented in Figure 1A, expression of both c-Src-R175***L*** and c-Src-W118***A***/R175***L*** showed a high degree of phosphorylation at Y418, which agrees with the stimulated tyrosine kinase activity of these mutants (Figure 1B). 

SFKs expression is associated with a different outcome in breast cancer patients [35]. Thus, we decided to determine the protein levels of several SFKs members in both cell models by WB. Expression of c-Src and Fyn was higher in SUM159 than in MDA-MB-231 cells, while the contrary was observed for c-Yes. Regarding Lyn protein, SUM159 cells showed higher Lyn A levels than MDA-MB-231, whereas the opposite situation was observed for Lyn B expression (Figure 1C). Expression of c-Src variants did not alter that of endogenous Src-kinases (data not shown). Therefore, we used two TNBC cell lines that exhibit a different expression pattern of SFKs, which better represent the variability of TNBC.

### 2.2. SH2 Domain of c-Src Is Important for In Vitro Breast Cancer Stem Cell-Renewal

**Figure 2:**
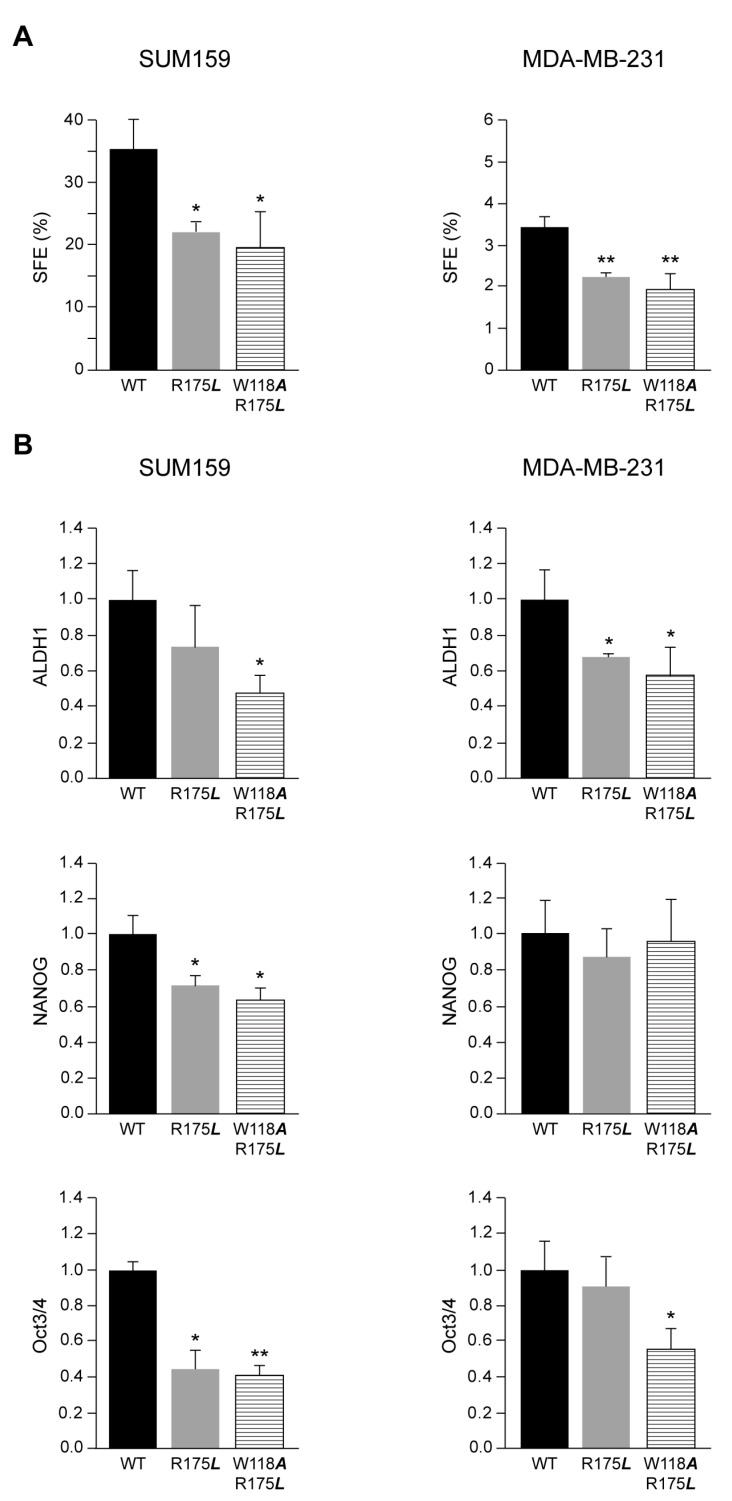
Role of c-Src variants in the self-renewal of breast cancer stem cells (BCSCs) derived from SUM159 and MDA-MB-231. The evaluation of self-renewal was determined by the sphere formation efficiency (SFE) in the enriched subpopulation of BCSCs derived from SUM159 and MDA-MB-231 (**A**). The SFE was measured at the third generation of mammospheres (see Materials and Methods). Each experiment was measured in triplicates (*n* = 3) and repeated three times. Results were expressed as a percentage of the mean ± standard deviation (SD). The statistical significance is referred to those obtained from c-Src-wt, * *p* < 0.5, ** *p* < 0.01. Quantitative analyses of stem cell markers ALDH1, NANOG, and Oct3/4 expression by WB in SUM159 and MDA-MB-231 cells conditionally expressing c-Src variants (**B**). Results represented data obtained from three independent WB (*n* = 3) using β-Actin as a loading control expressed as a percentage of the mean ± SD, and were referred to those obtained from cells expressing c-Src-wt considered as 1. The statistical significance is referred to cells expressing c-Src-wt, * *p* < 0.5, ** *p* < 0.01.

Within a tumor, there is a small portion of the tumor-mass (1–2%) derived from the stem-cell population that by mutations acquired tumorigenic properties. These breast cancer stem cells (BCSCs) are slow dividing and capable of regenerating a tumor upon transplantation in nude mice [35]. To determine the self-renewal capacity of the enriched population of BCSCs, we performed the mammosphere formation assay during three generations to define, at the third generation, the sphere formation efficiency (SFE, see Materials and Methods) [36,37,38]. We observed that SFE increased in all SUM159 and MDA-MB-231 cells expressing c-Src variants, indicating the enrichment of BCSCs population (Figures S1 and S2). Nevertheless, it should be noticed that SUM159 contained higher numbers of mammospheres than MDA-MB-231. We then analyzed the effect of c-Src variants described above (Figure 1A) on the BCSCs renewal ability of each cell line SUM159 or MDA-MB-231 expressing c-Src-mutants and compared it with the c-Src-wt. The functionality of SH2 and SH3 adapter domains appeared to be relevant for SFE, as both c-Src-R175***L*** and c-Src-W11A/R175L significantly reduced the self-renewal of the enriched population of MDA-MB-231 and SUM159-BCSCs (Figure 2A).

We have also found that the induction of SrcDN (c-Src-K295*M*/Y527*F*, which is devoid of catalytic activity but with functional SH2 and SH3 domains), a functional mirror image of c-Src-W118*A*/R175*L*, significantly reduced SFE in both SUM159 and MDA-MB-231. Furthermore, the endogenous c-Src function is required for SFE in MDA-MB-231 cells, as its conditional suppression [16] inhibited SFE (Figure S3). 

Altogether, the findings indicate that the three domains are necessary for self-renewal, as the alteration of only one of them reduced the sphere formation ability of both TNBC cells.

Consistent with the reduction of the enriched population of BCSCs induced by the expression of c-Src-R175L and c-Src-W118A/R175L c-Src mutants in both SUM159 and MDA-MB-231 cells, we analyzed the expression of ALDH1 by WB, a stem cell marker [36,37,38,39]. The results showed that in the MDA-MB-231 disruption of the functionality of c-Src SH2 domain by R175***L*** mutation inhibited the ALDH1 level as compared to c-Src-wt (Figure 2B and Figure S4). Disruption of the functionality of c-Src SH2 and SH3 domains by the double mutant c-Src-W118***A***/R175***L*** clearly reduced ALDH1 levels in either SUM159 or MDA-MB-231 (Figure 2B and Figure S4). Expression of NANOG and Oct3/4 was reduced in SUM159 cells expressing c-Src-R175L and c-Src-W118A/R175L mutants as compared to the expression of c-Src-wt (Figure 2B and Figure S4). In contrast, in MDA-MB-231, only the induction of c-Src-W118A/R175L mutant reduced the levels of Oct3/4, while none of the c-Src mutants altered the expression of NANOG (Figure 2B and Figure S4). Furthermore, the levels of NANOG and Oct3/4 were reduced in SUM159 and MDA-MB-231 cells following SrcDN expression or suppression of the endogenous c-Src (Figure S3).

Collectively, these results indicate that the SH2-c-Src domain is relevant for renewal of the enriched population of BCSCs in SUM159 and MDA-MB-231 cells. 

### 2.3. Role of Adapter Domains in Anchorage-Independent Growth

**Figure 3:**
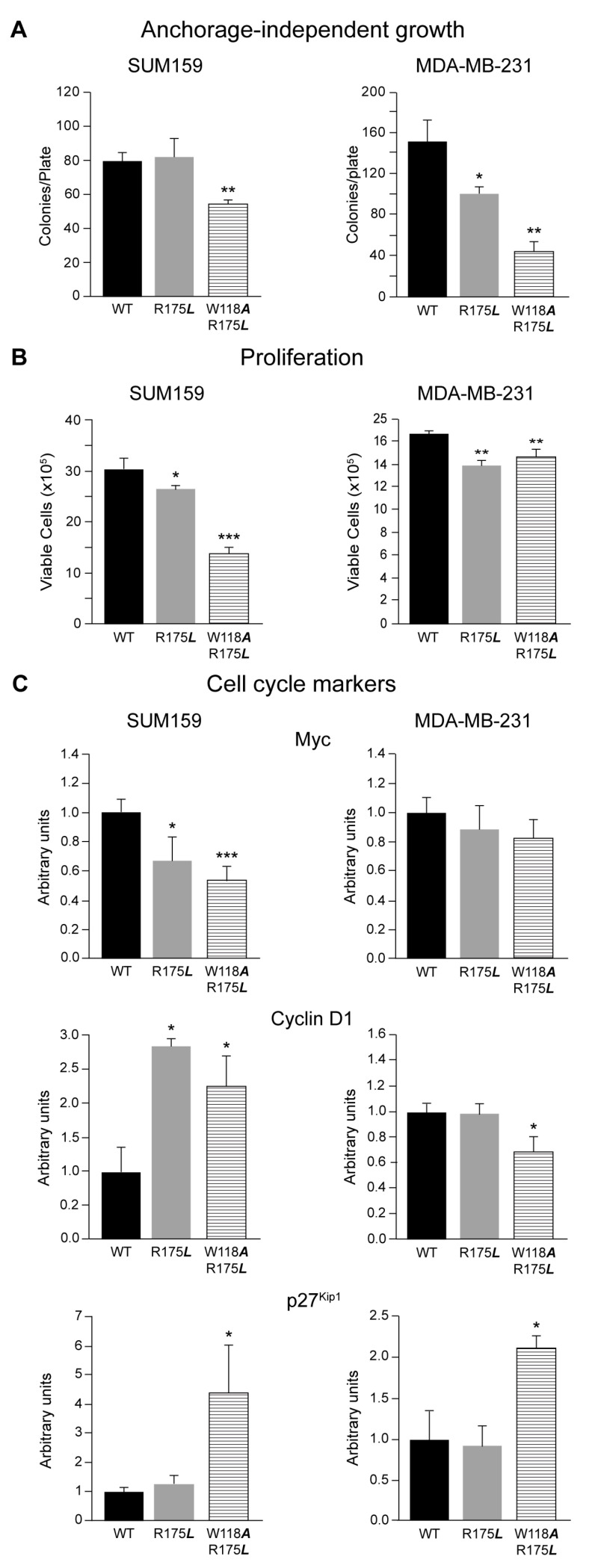
Modulation of anchorage-independent growth and cell proliferation by expression of c-Src variants in SUM159 and MDA-MB-231 cells. (**A**) Colony formation in soft-agar was employed to determine the effect of c-Src variants expression in SUM159 and MDA-MB-231 cells in anchorage-independent growth (see Materials and Methods). Each experiment was measured in triplicates (*n* = 3) and repeated three times. Results were expressed as the mean ± SD of the number of colonies/plate, * *p* < 0.5, ** *p* < 0.01. (**B**) Cell proliferation of SUM159 and MDA-MB-231 expressing c-Src variants was determined by the trypan blue exclusion assay (see Materials and Methods), * *p* < 0.5, ** *p* < 0.01, *** *p* < 0.001. (**C**) Quantitative analyses of cell proliferation markers Myc, cyclin D1, and p27 expression by WB in SUM159 and MDA-MB-231 cells conditionally expressing c-Src variants. Results represented data obtained from three independent WB (*n* = 3) using either β-Actin, α-Tubulin, or GAPDH as a loading control expressed as a percentage of the mean ± SD, and were referred to those obtained from cells expressing c-Src-wt considered as 1. The statistical significance is referred to cells expressing c-Src-wt, * *p* < 0.5, *** *p* < 0.001.

Anchorage-independent growth correlates with cellular tumorigenic and metastatic potential, a typical feature of in vivo TNBC aggressive phenotype. Thus, we analyzed the role of the SH2 adapter domain of c-Src in this event by determining cellular growth in soft agar. In SUM159 cells, induction of the c-Src-R175L mutant did not alter the colony formation as compared to the wild type. In contrast, mutation of the SH2 and SH3 domains together appeared to be relevant for colony formation in soft agar, as expression of c-Src-W118***A***/R175***L*** significantly reduced the number of colonies in the agar (Figure 3A and Figure S5). In MDA-MB-231 cells, Doxy induction of either c-Src-R175***L*** or c-Src-W118***A***/R175***L*** mutants significantly inhibited soft-agar colony formation, as compared to c-Src-wt (Figure 3A and Figure S5). Concurrently, these results support the role of the SH2-c-Src domain in anchorage-independent growth.

### 2.4. c-Src-SH2 Domain Modulates Cellular Proliferation 

Several data show the relevance of c-Src in cell proliferation and survival [1]. Therefore, we evaluated the effect of c-Src variants in SUM159 and MDA-MB-231 cell proliferation. In SUM159, induction of the c-Src-R175L and c-Src-W118*A*/R175L significantly reduced cell proliferation compared to c-Src-wt (Figure 3B). Similar results were obtained in MDA-MB-231 cells. Nevertheless, the reduction of cell proliferation observed in SUM159 was higher than in MDA-MB-231, which was modest (Figure 3B). When proliferation was analyzed considering cells without Doxy-induction as the control (Figure S6), since c-Src-wt expression did not alter proliferation, we observed similar results to those obtained considering c-Src-wt as the control. Cell cycle analyses by propidium iodide showed that in these mutants the number of cells in “Sub-G1” increased, and “G2-M” was reduced (Figure S7), which may help in understanding the proliferation differences. Analyses of cell cycle by pulse/chase with BrdU and propidium iodide in SUM159-Tet-On-c-Src-W118A/R175L showed an increased number of cells in the G1 phase upon induction of this mutant versus control (Doxy), a reduction of “S”, as well as in “G2/M”. MTT analyses of these cells showed a 45% reduction of metabolic active cells, which may be related to the number of viable and proliferating cells [40]. These results agree with those observed here (Figure 3B). Together, these results suggest that both c-Src-R175L and c-Src-W118A/R175L variants reduced proliferation with no signs of toxic effects.

We then analyzed by WB the levels of Myc, cyclin D1, and p27^kip1^ cell cycle makers (Figure 3C). The functionality of SH2-c-Src domain appeared relevant as induction of c-Src-R175L and c-Src-W118***A***/R175L in SUM159 reduced Myc expression. In contrast, in MDA-MB-231, no significant variations were detected upon induction of c-Src variants (Figure 3C). Interestingly, in SUM159 cells, expression of c-Src-R175***L*** and c-Src-W118A/R715L variants highly induced cyclin D1 (Figure 3C). In MDA-MB-231 cells, while c-Src-R175L did not modify cyclin D1 levels, the double mutant c-Src-W118A/R175L significantly reduced them. When the data were analyzed considering cells without Doxy induction as the control, the results showed the same tendency (Figure S6). The cyclin D1 gene regulation is complex and it varies between cell lines and experimental conditions. In rodent cells, it has been reported that Myc induces D1 in some cases while in others, Myc does not induce or even repress D1 (for review see [41]). Therefore, the downregulation of Myc observed in SUM159 cells may contribute to the upregulation of cyclin D1. 

Furthermore, regarding the cell cycle inhibitor p27^Kip1^, induction of both c-Src-R175*L* and c-Src-W118*A*/R175*L* mutants significantly increased its expression in SUM159 and in MDA-MB-231 (Figure 3C and Figure S6).

These results showed that SUM159 cells seemed to be more sensitive than MDA-MB-231 to the alteration of SH2-SH3 adapter domain functionality, as demonstrated by the effects induced by the expression of mutants on cell proliferation and cell cycle marker levels. Therefore, the kinase activity has an essential role in cell proliferation as the SH2 domain just partially modulates cell proliferation. 

### 2.5. Regulation of Cellular Migration and Invasion by c-Src Adapter Domains

**Figure 4:**
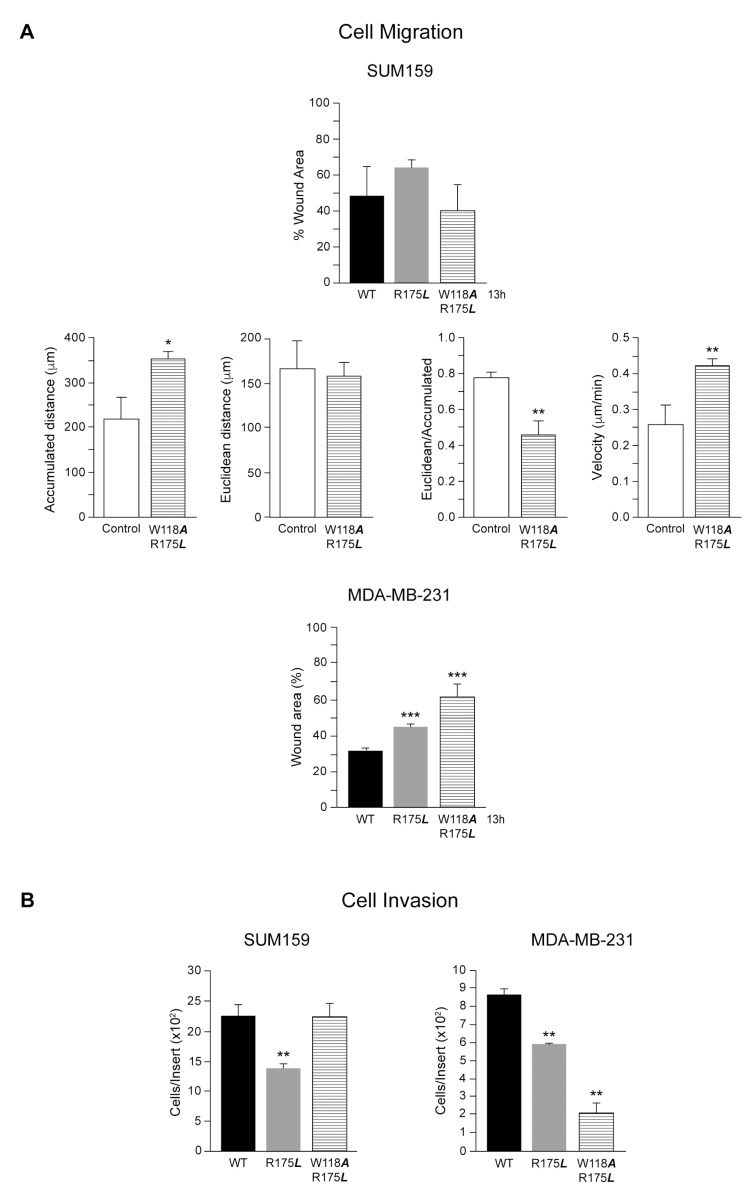
Effect of c-Src variants expression in SUM159 and MDA-MB-231cellular migration and invasion. Migration of cells was analyzed by wound-healing assays, and tracking analyses (*n* = 3), as described in Materials and Methods in SUM159 and MDA-MB-231 cells (**A**). Additionally, tracking analyses of W118A/R175L to determine the cell migration of at least 100 individual cells determine the Accumulated distance, Euclidean, and the Velocity of migration to evaluate the ration of Euclidean/Accumulated distance that define random migration. (**B**) The capability of cells to migrate through a layer of Matrigel was employed to determine cell invasion (see Materials and Methods) in both SUM159 and MDA-MB-231 conditionally expressing c-Src variants. The control value is similar to that in Figure 2. Results of three independent experiments (*n* = 3) were expressed as the mean ± SD. The statistical significance is referred to cells expressing c-Src-wt, * *p* < 0.5, ** *p* < 0.01, *** *p* < 0.001.

Cell migration is one of the essential steps of the metastatic cascade. We analyzed by wound-healing assays the effect of c-Src variants expression in SUM159 and MDA-MB-231 cell migration. In SUM159, Doxy induction of either c-Src -R175L or -W118A/R175L did not significantly alter migration as compared to c-Src-wt expression, and as shown by the lack of significant difference in the remaining wound-healing area after 13 h of migration between the control and the two variants (Figure 4A and Figure S9). When the analyses were made considering unstimulated c-Src variants (Doxy) as the control, it was observed that c-Src-wt inhibited cell migration in SUM159, while induced it in MDA-MB-231 (Figures S8–S10). Nevertheless, the results showed the same tendency as observed when the c-Src-wt expression was used as the control. However, looking at the recording videos of migration, we observed abnormal movements of SUM159 cells expressing the c-Src-W118A/R175L variant. Thus, we further analyzed the migration pattern of individual cells at the migration border by tracking the path of single cells. We found that only the expression of c-Src-W118***A***/R175***L*** variant in SUM159 cells caused random migration, as the ratio of Euclidean/Accumulated distances was significantly reduced, while the velocity of cell migration was increased, as compared to the control (Figure 4A and Figure S9). Consequently, SUM159 cells expressing the c-Src-W118A/R175L variant did not close the wound-healing area more or faster than cells expressing c-Src-wt or c-Src-R175L (Figure 4A). Indeed, they were moving randomly, not all in the direction to close the wound area, and at higher velocity than the other cells. In MDA-MB-231 cells, mutations affecting the SH2 domain functionality (R175L and W118***A***/R175L) reduced migration compared to c-Src-wt (Figure 4A and Figure S9). However, none of these mutations caused random migration in MDA-MB-231 cells. 

The oncogenic potential of c-Src in tumor cells is pleiotropic and controls cytoskeletal-linked events, such as extracellular matrix-adhesion, migration, and invasion. We previously showed that the catalytic activity of this proto-oncogene is involved in invasion and migration [15]. Now, we analyzed whether the SH2 adapter domain of c-Src is involved in the regulation of SUM159 and MDA-MB-231 cellular invasion. In SUM159, expression of c-Src-R175*L* variant inhibited the number of invading cells, while the double SH2/SH3 mutant c-Src-W118A/R175L, was unable to modify the invasiveness of SUM159 cells, as compared to the c-Src-wt (Figure 4B). In contrast to SUM159, expression of c-Src -R175L and -W118A/R175L significantly inhibited cell invasion in MDA-MB-231 in comparison to c-Src-wt (Figure 4B). When the analyses were made considering unstimulated c-Src variants (Doxy) as the control, it was observed that c-Src-wt did not alter invasiveness in SUM159, while induced it in MDA-MB-231 (Figure S8). Nevertheless, the results showed the same tendency as observed when the c-Src-wt expression was used as the control.

The growth factor or integrin stimulation induces Fak autophosphorylation on Y397, generating a high affinity binding site for the Src SH2 domain [20,21], which in turn phosphorylates Fak at several tyrosine residues allowing the activation of multiple signaling pathways [42]. The association of Src with Fak controls the turnover of focal adhesion complexes, which are involved in cell motility, migration, and invasion [39]. These processes involve the dynamic control of protein associated with focal adhesion complex, among them, Fak, Paxillin, Caveolin 1, etc., due to, at least in part, their phosphorylation/activation [16,42,43]. We then analyzed the degree of activation/phosphorylation of these proteins involved in cell migration and invasion by WB. In SUM159, expression of c-Src-W118A/R175L increased Caveolin 1 levels (Figure S11A), while only c-Src-R175***L*** increased pY14-Caveolin 1. Paxillin expression remained constant upon induction of c-Src variants, albeit pY118-Paxillin/Paxillin augmented in c-Src-R175L expressing SUM159 cells (Figure S11A). Fak expression was constant, whereas pY397 diminished upon induction of c-Src-R175L and c-Src-W118A/R175L compared to c-Src-wt. Phosphorylation of Fak at Y576 was significantly reduced in cells expressing c-Src-R175L, while it was increased by c-Src-W118A/R175L, as compared to c-Src-wt (Figure S11A). In MDA-MB-231, expression of Caveolin 1 remained constant for all c-src variants expressing cells (Figure S11B). The mutants c-Src-R175L and c-Src-W118A/R175L have an open conformation, consequently, they highly increased the activation of Caveolin 1 (Figure S11B). Expression of c-Src mutants did not alter the levels of Paxillin protein, while the activation of Paxillin (ratio p118Y-Paxillin/Paxillin) was surprisingly inhibited in MDA-MB-231 expressing c-Src -R175L and -W118A/R175L, as compared to c-Src-wt (Figure S11B). Fak protein levels were unaltered in any of the MDA-MB-231 cell lines expressing c-Src variants (Figure S11B). Conversely, Fak autophosphorylation at Y397 was increased by expression of W118A/R175L. The specific activity of Fak (pY576-Fak/Fak) was not augmented in all c-Src mutants expressed in MDA-MB-231 (Figure S11B) as compared to c-Src-wt. When the results of WBs were analyzed considering non-induced conditions for c-Src variants as the control (Doxy conditions), the results showed the same tendency (Figures S12 and S13). When all the c-Src variants expressing SUM159 and MDA-MB-231 cells were analyzed together in a single WB, the results showed that expression of total c-Src was higher in SUM159 than in MDA-MB-231, supporting the data from Figure 1B, as it was observed for total Fak. Changes in pY397-Fak were not evident in any of the two cell lines. In contrast, phosphorylation of Fak by c-Src at Y576 increased upon expression of the mutants in both cell lines. However, results from triplicate experiments (Figures S11–S13) showed inhibition of pY576-Fak in SUM159 expressingc-Src-R175L. Possibly, this discrepancy is due to the fact that the analysis of Figure S14 represents a single sample, while data from Figures S11 and S12 represented the average of three different samples. Similarly, while the levels of total Akt practically unchanged in SUM159 and MDA-MB-231 cells due to the expression of c-Src variants, the extent of pS473-Akt was increased upon induction of all the c-Src variants (Figure S14).

We have also analyzed the effect of c-Src mutants in the cellular distribution of pY14-Caveolin 1 and pY418-Src in both SUM159 and MDA-MB-231 cells by confocal microscopy. In c-Src-wt or in c-Src-R175***L*** expressing SUM159 cells, pY14-Caveolin 1 and pY418-Src co-localized at the focal adhesion sites (Figure S15), whereas in those expressing c-Src-W118***A***/R175***L***, distribution of pY418-Src did not fully co-localize with pY14-Caveolin 1 at focal adhesion sites (Figure S15 basal layer, and Figure S15 upper layer). However, if we focus at higher levels, we find that in both mutant expressing SUM159 cells pY14-Caveolin and pY418-Src decorated caveolae (spherical structures within the cellular cytoplasm) (Figure S15). 

Confocal microscopy analyses at the basal layer of MDA-MB-231 overexpressing c-Sr-wt and -R175***L*** showed a distribution of pY14-Caveolin 1/pY418-Src at the adhesion areas. The intracellular accumulation of pY418-Src was also detected at perinuclear areas. In cells expressing c-Src-W118A/R175L, the focal adhesion was not clearly displayed (Figure S15). The distribution of pY14-Caveolin 1/pY418-Src in MDA-MB-231-c-Src-R175L at the upper layer showed their co-localization at the perinuclear region, and in the cytoplasm where they decorated some vacuolar structures, as observed in SUM159. Similar to SUM159, in MDA-MB-231-c-Src-W118***A***/R175***L***, the vacuolar structures were also observed but to a much lesser extent (Figure S15).

### 2.6. SH2-c-Src Directed Aptamers Reduced Cell Proliferation

**Figure 5:**
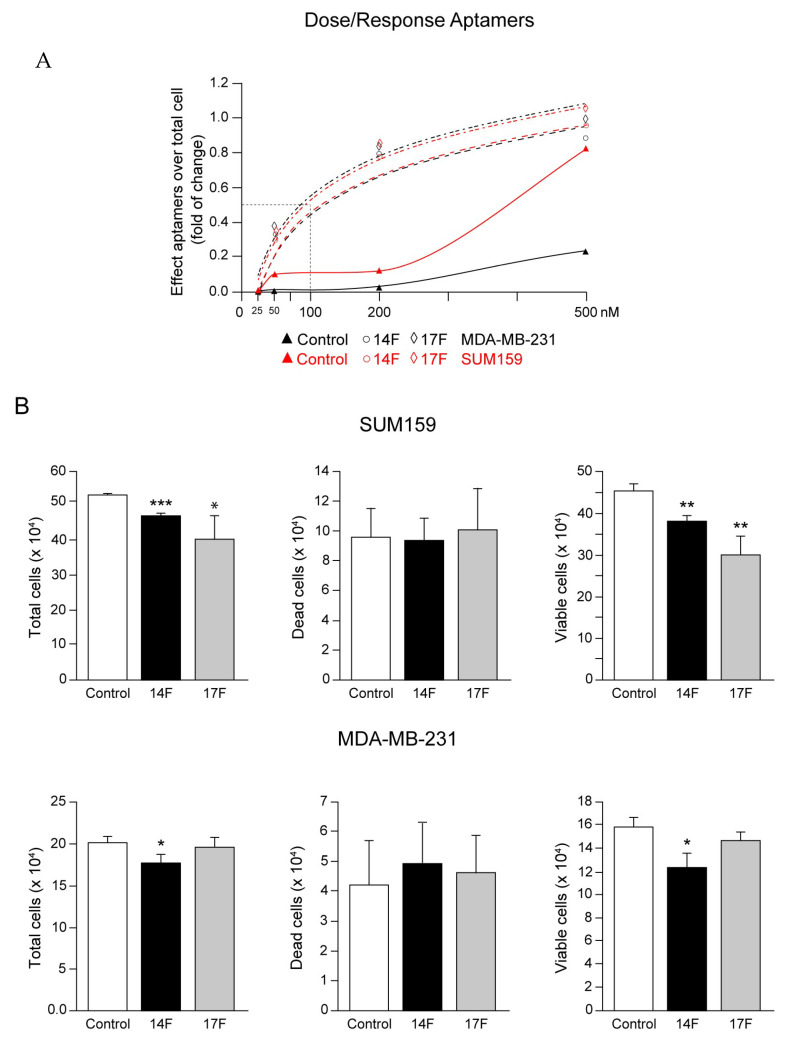
Dose/response of SH2-c-Src directed aptamers in SUM159 and MDA-MB-231 cells and evaluation of cell proliferation. (**A**) Control aptamer (38xAG) and SH2-c-Src directed aptamers 14F and 17F were transfected at 25, 50, 200, and 500 nM to SUM159 and MDA-MB-231and the IC50 values were determined as described in Material and Methods (*n* = 3). (**B**) Control and SH2-c-Src directed aptamers 14F and 17F were transfected at 100 nM to either SUM159 or MDA-MB-231 and, 72 h later, the number of total, dead, and viable cells were determined by the trypan blue exclusion method (see Materials and Methods). Each experiment was measured in triplicates (*n* = 3) and repeated three times. Results were expressed as a percentage of the mean ± SD, * *p* < 0.5, ** *p* < 0.01, *** *p* < 0.001.

The 14F and 17F aptamers directed to SH2-c-Src were designed and selected as described (Materials and Methods, and in the extended methods in Supplementary Information). The analyses of aptamers have been referred to the aptamer control (containing 38xAG, see Material and Methods), as in preliminary studies no differences were detected in cell proliferation of either SUM159 or MDA-MB-231 cells when compared to the transfection of the control (38xAG) and mock (empty transfection) [40]. We then determined the IC50 concentration for each aptamer in SUM159 and MDA-MB-231 cells by considering the total number of cells after the treatment with different concentrations of the aptamers (0, 25, 50, 200, and 500 nM), taking as 0% the concentration of aptamers with no effect and as 100% the effect at the maximal concentration of the aptamers for each cell line (see Materials and Methods). The results were graphically represented (Figure 5A). The IC50 values for 14F (117.1 and 141.7 nM) and 17F (94.3 and 97.0 nM) for SUM159 and MD-MB-231, respectively, were slightly different. Nevertheless, we decided to perform all the experiments at 100 nM for each aptamer at both cell lines. We determined the effect of SH2-c-Src directed aptamers (100 nM) in cell proliferation using the trypan blue exclusion method, allowing us to evaluate the total number of cells, the dead cells, and the living cells 72 h after transfection. The results showed (Figure 5B) that in both cell lines the number of dead cells was similar for all aptamers control (aptamer control), 14F and 17F, indicating that at this concentration they were not cytotoxic (Figure 5B). The 14F and 17F aptamers reduced the total cell number, as well as the number of viable cells in SUM159, while in MDA-MB-231 only the 14F caused a significant reduction of total and viable cells, as there is no significant reduction in viable cells in the MDA-MB-231 cell line with the 17F aptamer. 

Then, we analyzed the levels of Myc, cyclin D1, and p27^Kip1^ in SUM159 and MDA-MB-231 cells treated with 14F and 17F aptamers. As compared to the aptamer control considered as 1, these aptamers reduced the expression of Myc and cyclin D1, while they increased those of p27^Kip1^ in both SUM159 and MDA-MB-231 cells (Figure S18).

### 2.7. Role of 14F and 17F in Cell Migration and Invasion 

**Figure 6:**
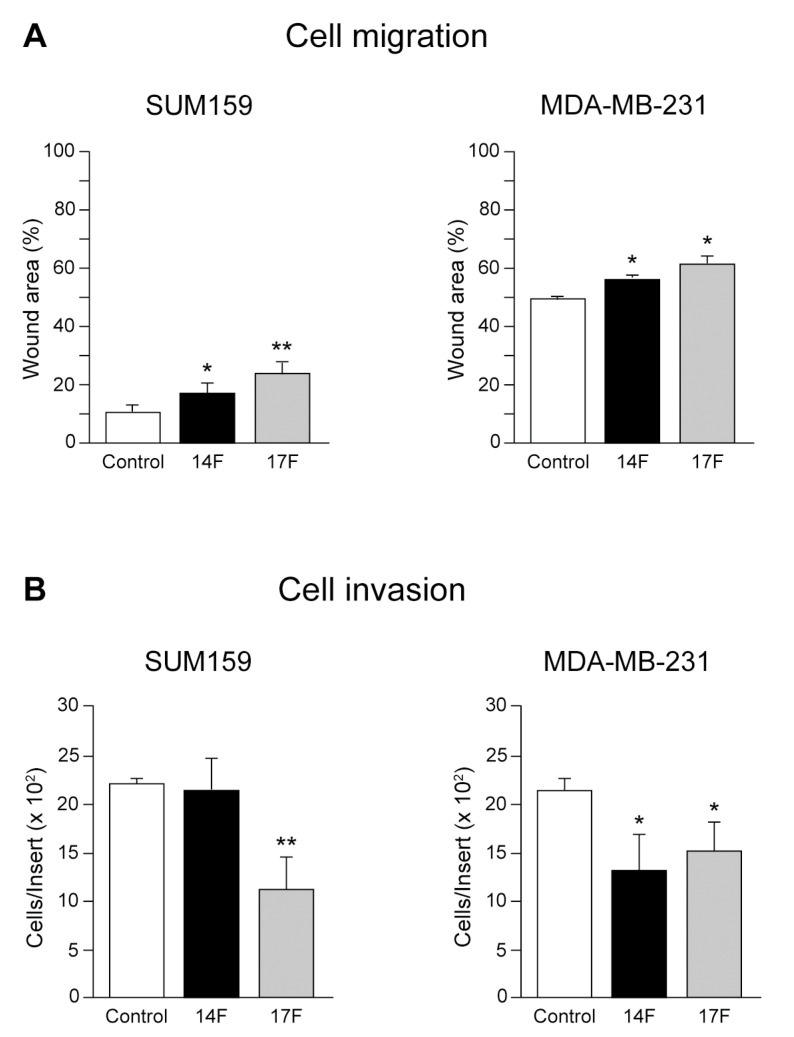
Role of SH2-c-Src directed aptamers in SUM159 and MDA-MB-231 cells to evaluate cellular migration and invasion. Wild type SUM159 and MDA-MB-231 cells were transfected with either control, 14F or 17F aptamers at 100 nM and cell migration (**A**) and invasion (**B**) were determined as described in Materials and Methods. Each experiment was measured in triplicates (*n* = 3) and repeated three times. Results were expressed as a percentage of the mean ± SD, * *p* < 0.5, ** *p* < 0.01.

As cell migration and invasion are steps required for TNBC metastasis, we evaluated the relevance of the SH2 -c-Src domain in the regulation of these events by blocking its functionality in SUM159 and MDA-MB-231. We first observed that MDA-MB-231 cells migrated significantly less than SUM159, as the wound area after 13 h of migration was bigger. In either of the cell lines, the aptamers significantly reduced migration, as both 14F and 17F had a bigger wound area at the end of the experiments compared to the control (Figure 6A, Figures S16 and S17).

As for cell invasion, while only the 17F aptamer clearly reduced invasiveness in SUM159 cells, both 14F and 17F inhibited cell invasion of MDA-MB-231 cells (Figure 6B). We analyzed the effects of 14F and 17F aptamers on the expression of c-Src, pY418-Src/C-Src, Fak, pY397-Fak, Caveolin 1, pY14-Caveolin 1/Caveolin 1, Paxillin, and pY118-Paxillin/Paxillin in both SUM159 and MDA-MB-231 cells by WB considering the aptamer control as 1. In SUM159 and MDA-MB-231 cells, expression of c-Src and the activated form were unmodified, as also observed for Fak and auto-phosphorylated (Figure S18). In contrast, Caveolin 1 levels were reduced by aptamer 14F and increased by aptamer 17F, whereas pY14-Caveolin 1/Caveolin 1 was reduced by both aptamers in SUM159, but not in MDA-MB-231. Paxillin levels were unaltered by either of these aptamers, although the pY118-Paxillin/Paxillin ratio was significantly reduced by both 14F and 17F in both SUM159 and MDA-MB-231 cells (Figure S18). These results are different to those observed in Figure S11, as employing aptamers were blocking the functionality of the SH2 domain (Figure S18), while through the other experimental approach the induction of expression of c-Src variants was achieved. 

Considering that migration and invasion are required for the metastatic process, these results support the experiments carried out with the conditional expression of c-Src variants and indicated that the SH2 domain is relevant for c-Src functionality.

## 3. Discussion

The SFKs control many signaling pathways involved in the regulation of several cellular processes. Thus, the deregulation of their functionality is associated with tumors, including breast cancer [1,7,44]. Here, we studied the relevance of the SH2 adapter domain of c-Src in two TNBC cell lines SUM159 and MDA-MB-231. Several scientific reports showed that, even though the two cell lines share several common characteristics, as both are considered Basal-Mesenchymal TNBC cell lines with a spindle phenotype, SUM159 (primary breast adenocarcinoma) has mutations in HRAS and PIK3CA (https://web.expasy.org/cellosaurus/CVCL_5423), while MDA-MB-231 (pleural effusion) has deletions in p14ARF, p16, and CDKN2, and mutations in the KRAS, BRAF, and TERT promoter (https://web.expasy.org/cellosaurus/CVCL_0062), supporting the heterogeneity observed in TNBC [12,45,46,47,48].

Our analyses of SFKs expression in SUM159 and MDA-MB-231 cells showed that c-Src, Fyn, and Lyn A were expressed at higher levels in SUM159 than in MDA-MB-231, while the opposite occurred for Lyn B and Yes.

To determine the relevance of the SH2-c-Src domain we followed two independent and complementary approaches, the conditional expression of c-Src variants with inactivating point mutations affecting SH2 functionality (R175L, W118A/R175L), and the transfection of two different aptamers directed to the SH2-c-Src domain, that interacted and then blocked the SH2-c-Src function in SUM159 and MDA-MB-231

It is well established that c-Src increased its expression/activity as the tumor progresses [1,5,7]. Our results also indicated that the c-Src played a relevant role in the renewal of the enriched population of BCSCs. The role of Src in the maintenance of these cells was previously observed in MCF7 [38], and here in SUM159 and MDA-MB-231 cells by the conditional expression of the dominant negative form of c-Src (SrcDN), as well as in MDA-MB-231 that conditionally have c-Src suppressed. Indirectly, expression of miR205 in SUM159, which inhibits SFKs members expression, suppressed SUM159 BCSCs renewal and stem cell markers [37]. Moreover, selective inhibitors of SFKs tyrosine-kinase activity also have an inhibitory effect in BCSCs renewal. In SUM159, resistant to paclitaxel, Dasatinib causes epithelial differentiation and enhances sensitization to paclitaxel, and a combination of both compounds reduces stem cell renewal and synergizes to diminish the viability of paclitaxel-resistant SUM159 cells [49]. In high-grade serous ovarian cancer cells, co-treatment of Saracatinib (SFKs inhibitor, AZD0530) and selumetinib (MEK inhibitor, AZD6244) reduced SFE and ALDH1 positive cells and, in vivo the loss of tumor-initiating cells following serial tumor xenografting [50]. Our results showed that the inactivating mutation of the SH2 and SH3 domains R175***L*** and W118***A***/R175***L*** significantly reduced BCSCs renewal in both cell lines, supporting the relevance of the adapter domains in the renewal of the tumor initiating cells. In primary PDAC cultures, established from patient-derived xenografts with Dasatinib or PP2 reduced the clonogenic, self-renewal, and tumor-initiating capacity of PaCSCs, which we attribute to the downregulation of key signaling factors such as p-FAK, p-ERK1-2, and p-AKT [51].

The anchorage-independent growth, which characterized tumor cells, showed the discrepancy between SUM159 and MDA-MB-231 cells. While in SUM159 cells only the suppression of both SH2 and SH3 functionality reduced it, in MDA-MB-231 cells, both c-Src-R175L and c-Src-W188A/R175L diminished colony formation in soft-agar as compared to c-Src-wt. Suppression of endogenous c-Src in MDA-MB-231 cells significantly reduced anchorage-independent growth [16]. Likewise, specifically silencing c-Src and not Yes or Fyn inhibited soft-agar colony formation in MDA-MB-231, MDA-MB-436, and SKBR3 [52]. Furthermore, SFKs catalytic activity inhibition or stable transfection of catalytically inactive c-Src into MDAMB-468 and MCF7 reduced the colony formation ability [53]. In addition, inhibition of SFKs expression by miR205 in SUM159 significantly diminished the anchorage-independent growth [37]. Inhibitors of SFKs catalytic activity such as Dasatinib inhibits soft-agar colony formation in BxPC3 and PANC1 pancreatic cancer cells [54]. Therefore, the SH2-c-Src domain appeared relevant for MDA-MB-231 breast cancer cells in the anchorage-independent growth.

Cell proliferation was also influenced by SH2 functionality, as it was reduced upon induced expression of c-Src-R175L and c-Src-W118A/R175L variants as compared to c-Src-wt, as it was Myc expression in SUM159. On the contrary, we observed that in MDA-MB-231, inhibition of cell proliferation was not linked to alteration in the levels of Myc. Inhibition of SFKs tyrosine-kinase activity clearly blocks cell proliferation in MDA-MB-231 cells [15]. Interestingly, while Myc expression was reduced, cyclin D1 increased in response to the expression of c-Src-R175L and c-src-W118A/R175L in SUM159 cells. In this context, in rodent cells, it has been reported that Myc induces D1 in some cases, while in others, Myc does not induce or even repress D1, supporting the concept of the complex regulation of cyclin D1 gene (for review see [41]). Thus, the downregulation of Myc observed in SUM159 cells may contribute to the upregulation of cyclin D1.

Numerous studies show that the catalytic activity of SFKs is important for migration and invasion of tumor cells [6,15,16,22,55,56,57]. Induction of Fak autophosphorylation by the growth factor or by integrins facilitates its interaction with the SH2 domain of c-Src, opening c-Src conformation and, consequently, increasing its tyrosine kinase activity. Then, c-Src phosphorylates Fak at other sides, and facilitates the interaction/activation of other signaling molecules [1,7,20,21]. The complex Src/Fak phosphorylates/activates several focal adhesion proteins involved in migration and invasion [16,39,42,43]. In addition, our results showed that the SH2-c-Src domain was also relevant for modulation of invasion in both TNBC cell lines. Interestingly, they also revealed in SUM159 cells that altering the functionality of both SH2 and SH3 c-Src domains (c-Src-W118A/R175L variant) caused migration to occur in a random manner, as cells had high motility but they did not close the wound healing area. In HT1080 fibrosarcoma, overexpression of PEAK1 kinase, which is phosphorylated/activated at Y665 by SFKs, causes random migration and elevates cell invasion [58]. Consistently, in SUM159 cells, increased migration was associated with the activation of focal adhesion proteins caveolin1, paxillin, and Fak which were increased by overexpression of c-Src-W118A/R175L variant. In contrast, the c-Src-R175*L* reduced pY576-Fak/Fak. Confocal-microscopy analyses of the cellular distribution of activated Src (pY418-Src) and caveolin 1 (pY14-Caveolin 1) in SUM159 cells expressing the c-Src-R175L mutant at the basal layer showed their colocalization at the focal adhesion sites, as observed in SUM159-c-Src-wt. Interestingly, at the upper layer of analyses both c-Src -R175***L*** and -W118A/R175L variants showed co-decoration of caveolae-like structures as compared to c-Src-wt. As mentioned above, SUM159 and MDA-MB-231 cells though they share a good number of common properties, also show some differences. Nevertheless, results showed that the SH2-c-Src domain played an important functional role in SUM159 and MDA-MB-231 TNBC cells. 

To support these data, we approached this study by a different and complementary method. We designed two different aptamers directed to interact with the SH2-c-Src domain. The results showed that at a dose around the IC50 concentration both aptamers significantly inhibited the proliferation of SUM159 and MDA-MB-231, without inducing apoptosis, as the number of dead cells was unaltered. In agreement with these observations, the expression of Myc and cyclin D1 were reduced, while p27Kip1 levels were augmented. As observed for cells expressing c-Src-R175***L***, these aptamers inhibited migration and invasion in both TNBC cells. The aptamers design to bind to MNK1, which controls the eIF4E function by phosphorylation, significantly inhibits proliferation and migration of MDA-MB-231 [30]. The AS1411 aptamer directed to nucleolin induces blc-2 mRNA instability, reduces cell growth by causing cytotoxicity in MCF7 and MDA-MB-231 [59]. This aptamer has been tested in different tumors including glioma, renal cell carcinoma [31].

The results obtained indicated that the functionality of SH2-c-Src domain is important for the potential tumorigenicity of SUM159 and MDA-MB-231 cells as the inactivating point-mutation of this domain inhibited the biological functions required for the TNBC cell. Similarly, aptamers directed to the SH2-c-Src domain also significantly reduced the performance of these TNBC cells. 

## 4. Materials and Methods

### 4.1. Antibodies and Reagents

Table S1 contains the antibody information. The chemical reagents and enzymes used were of analytical grade and purchased from Thermo-Fisher (Waltham, MA, USA), Roche (Basel, Switzerland), Corning (Merck, Darmstadt, Germany**)**, PeproTech (London, UK), PAA Laboratories GmbH (Cölbe, Germany), Bio-Rad (Hercules, CA, USA), GE Healthcare and Sigma-Aldrich/Merck (Merck, Darmstadt, Germany). 

### 4.2. Cell Lines and Culture

MDA-MB-231 (HTB-26) was from ATCC, and SUM159PT (CVCL-5423) [60] was provided by Dr. G. Dontu [61]. Cell lines were mycoplasma free and authenticated by the short-tandem-repeat analysis (GenePrintR 10 System from Promega (Madison, WI, USA), and GeneMapper v3.7 STR profile analysis software, Life Technologies, Carlsbad, CA, USA) (see Supplementary Information). Profiles were checked against public databases ATCC and DSMZ. MDA-MB-231 was maintained in DMEM, 5% FCS, 2 mM glutamine, 100 IU/mL penicillin, and 100 μg/mL streptomycin. SUM159 was cultured in Ham’s F12, 5% FCS, 5 μg/mL insulin, 1 μg/mL hydrocortisone, 2 mM glutamine, 100 IU/mL penicillin, and 100 μg/mL streptomycin.

Generation of SUM159PT-Tet-On-c-Src- and MDA-MB-231-Tet-On-c-Src -wt, -R175L, - and -W118A/R175L, was carried out as described [16], and grown in the presence of 3 μg/mL blasticidin, 100 μg/mL zeocin to maintain the plasmid selection of cells expressing c-Src-wt and c-Src-R175L or with 3 µg/mL blasticidin, 3 μg/mL hygromycin for the selection of cell expressing c-Src-W118A/R175L.

The wild type (wt) and c-Src variants used in these experiments were from a chicken origin [1,4,19]. The BLASTp comparative analysis of c-Src protein sequences between *Homo Sapiens* (Protein Accession Number: P12931.3) and *Gallus-Gallus* (Protein Accession: P00523.4) resulted in over 94% identity at the amino acid sequences [33]. Since the EC10 mouse monoclonal antibody (Millipore, #05-185) specifically recognizes chicken c-Src, it was possible to determine c-Src variants in the presence of the endogenous human c-Src of SUM159 and MDA-MB-231 cells.

### 4.3. Mammosphere Cultures

Single cell suspensions of adherent cultures were plated in 6-well ultralow attachment plates (Falcon, Corning Life Science, Merck, Darmstadt, Germany) at 2 × 10^3^ cells/well. Mammosphere cultures were maintained in serum-free DMEM/F12 media (1:1), B27 (1:50), EGF (20 ng/mL) and bFGF (20 ng/mL), insulin (5 µg/mL), hydrocortisone (5 µg/mL). After 10 days, cells were pipetted up and down to eliminate cellular aggregates and mammospheres (sphere-like structures with diameter ≥ 50 μm) were clearly detected by the optical phase contrast microscope (Nikon-Eclipse TS100, 4× magnification). Cultures were then trypsinized to induce mammosphere dissociation to single cells, which were seeded again for mammosphere formation. The experiment ended at the third generation of mammosphere formation. Sphere forming efficiency (SFE) was then calculated as the number of spheres formed per number of seeded cells and expressed as % means ± SD, as described [37,38]. 

### 4.4. Anchorage-Independent Growth

Cells were resuspended in a warmed solution of 0.3% agarose in a complete medium and seeded at 10^5^ cells/60 mm dishes with a bottom layer of 0.5% agarose. Cells were re-fed every 72 h with a complete medium (300 µL/dish). At the 10-day growth, plates were stained with 0.5 mL of 0.005% crystal violet/water for 1 h and colonies with diameter ≥ 0.1 mm from 4–5 fields/plate were counted, as described [37].

### 4.5. Cell Proliferation

Cell proliferation was evaluated by counting viable cells performing a Trypan blue (Sigma-Aldrich) exclusion assay. Cells were seeded at 3 × 10^5^ cells/60 mm dishes, 72 h later they were trypsinized, cells were pelleted and resuspended in a culture medium, mixed with a 0.4% Trypan blue/PBS solution (1:1), loaded on a hemocytometer, and Trypan blue-negative (viable cells) and Trypan blue-positive cells (dead cells) were counted. 

### 4.6. Cell Migration

Cells were seeded in a complete medium in a 6-well plate and grown to confluence. The monolayer was scratched with a 200 µL micropipette tip, and washed with a fresh medium to remove floating cells. A complete medium was added to the cultures, and photomicrographs were taken every 30 min with a Microscope Cell Observer Z1 system (Carl Zeiss AG) equipped with a controlled environment chamber and Camera Cascade 1 k to monitor the wound closure. Migration was quantified using the wound-healing tool ImageJ, as described [15,37]. Tracking of cell migration was carried out in 100 cells/assay using the “manual-tracking” from the ImageJ program together with the “chemotaxis and migration tool”. 

### 4.7. Invasion Assay

Invasiveness was determined as described [15]. Briefly, cells were seeded in a serum-free medium on the upper chamber of cell culture inserts of 24-well plates (8 μm-pore PET membranes, BD) coated with Matrigel^TM^ (5 × 10^4^/well/200 μL). The lower chamber was filled with 600 μL of 20% FBS; 22 h later, after removing the cells on top of the inserts, those on the lower surface were fixed with methanol, nuclei stained with DAPI, and mounted on slides with a Prolong antifade-reagent. Filters were observed with a Plan 20×/0.50 objective of an axiophot fluorescence microscope (Zeiss, Oberkochen, Germany) equipped with an Olympus DP70 digital camera. DAPI-stained nuclei were counted. 

### 4.8. Western Blot Analysis 

Cell lysates preparation and Western Blot (WB) analyses were carried out, as previously described [37]. Briefly, cells were lysed at 4 °C with a lysis buffer (10 mM Tris–HCl (pH 7.6), 50 mM NaCl, 30 mM sodium pyrophosphate, 5 mM EDTA, 5 mM EGTA, 0.1% SDS, 1% Triton X-100, 50 mM NaF, 0.1 mM Na_3_VO_4_, 1 mM PMSF, 1 mM benzamidine, 1 mM iodoacetamide, and 1 mM phenantroline). Cell lysates were obtained by centrifugation at 21,380× *g* for 30 min at 4 °C; the protein concentration in the supernatant was determined by the BCA protein assay (Pierce, Rockford, IL, USA), and lysates were adjusted to equivalent concentrations with a lysis buffer. Aliquots of 30 µg of total cell lysates were then separated on SDS–PAGE. Proteins were transferred to PVDF membranes that were blocked 1 h at room temperature with 5% non-fat milk in TTBS (TBS with 0.05% Tween-20) or 5% BSA in TTBS for phosphoproteins. Incubation with primary specific antibodies was carried out overnight at 4 °C, and horseradish peroxidase-conjugated secondary antibodies in a blocking solution for 1 h at room temperature. Immunoreactive bands were visualized by the ECL kit. 

### 4.9. Aptamers Design and Selection

The SH2 and SH3 domains of c-Src cloned into GST [62] and expressed in *E. coli* were purified from the soluble fraction by glutathione-resin affinity chromatography (Genescript, Piscataway, NJ, USA) as described [30]. Aptamers selection, cloning and sequencing, and secondary ssDNA structure prediction, as well as an enzyme-linked oligonucleotide assay (ELONA) methodology was previously described [30]. The aptamers employed were: 1. Control containing 38xAG, as described [30]; ApSH2.F14: GCGGATGAAGACTGGTGTAGACAATGGATACTCCCGCCACCTCCTCCCCCG CCCCCCCGCCCTAAATACGAGCAAC; ApSH2.17F: GCGGATGAAGACTGGTGTGCGGTGGT GGGTTGGGTGGGTGGGTTTGCGGGTTGCGTTGGCCCTAAATACGAGCAAC. Please see the extended method in the Supplementary Information for details.

### 4.10. Aptamers Transfection and IC50

SUM159 and MDA-MB-231 cells were seeded in 24 multi-well plates (10^4^ cells/well/500 μL) in their corresponding culture media without antibiotics; 24 h later, cells were washed twice with their serum and antibiotic-free corresponding media. Then, the cells were incubated in 400 μL of culture media without antibiotics and 100 μL of the transfection mixture: 1. 0.25, 0.5, 2, or 5 μL of each aptamer, corresponding to 25, 50, 200, or 500 nM, in 49.75, 49.5, 48, or 45 μL of culture media; 2. 1.25 μL of DharmaFECT-4 (Thermo-Scientific) in 48.75 μL culture of media, following the manufacturer’s manual; 8 h later, the cells were extensively washed with a culture media without antibiotics, then incubated for an additional 40 h and then analyzed as previously described in the cell proliferation assay section. The IC50 for each aptamer was determined considering the total number of SUM159 or MDA-MB-231 cells after the treatment with different concentrations of the aptamers. To this end, the untreated cells were considered as 0% (without effect) and 100% the effect at the maximal concentration of the aptamers for each cell line. Then, the obtained data were graphically represented employing the mathematical formula for the logarithmic trendline calculated with Excel to obtain the IC50 for each aptamer. Then, the IC50 values of each aptamer were used to determine their effects in cell proliferation, migration, and invasion of SUM159 and MDA-MB-231 cells, as previously described. 

### 4.11. Statistical Analyses

Mean values, standard deviation, and statistical significance between data from the two different experimental conditions (±Doxy) were determined by the two-tail Student *t*-test. Data were normalized to the activity of the c-Src-wt variant for each cellular assay.

## 5. Conclusions

Our results conclude that the SH2-c-Src domain functionality is relevant for the potential tumorigenicity of SUM159 and MDA-MB-231, as the inducible expression of c-Src with the unfunctional SH2-c-Src domain inhibited the renewal of the enriched BCSCs cells, as well as other relevant functionalities in these TNBC cells. Similarly, the aptamers directed to the SH2-c-Src domain also significantly reduced the performance of these TNBC cells. Therefore, using a combination of SH2-c-Src functional inhibitors with those directed to the tyrosine kinase activity should be able to fully block the c-Src functionality and, consequently, could be therapeutically effective in the breast cancer treatment. Furthermore, as c-Src is also involved in other types of tumors (pancreas, colorectal, lung, etc.) [1,7,18,43,63,64], our results could eventually be extrapolated to these other pathologies. 

## Data Availability

The data presented in this study are available in Cancers-1076808 and in its Supplementary Materials.

## Supplementary Materials

The following are available online at https://www.mdpi.com/2072-6694/13/3/462/s1. 1. Supplementary Materials and Methods: 1. Purification and selection of aptamers for the SH2 domain of c-Src (extended method); 2. Immunofluorescence by lasers-canning confocal microscopy; 2. Supplementary Figures: Figure S1: Analyses of sphere formation efficiency (SFE); Figure S2: Images of mammospheres of SUM159 and MDA-MB-231 cells expressing c-Src variants; Figure S3: Effect of conditional expression of SrcDN in SUM159 and MDA-MB-231 cells or suppression of endogenous c-Src in MDA-MB-231 on SFE; Figure S4: Western blot analyses of ALDH1, NANOG, and Oct3-4 from mammospheres of SUM159 and MDA-MB-231 cells expressing c-Src variants; Figure S5: Soft-agar colonies from SUM159 and MDA-MB-231 cells expressing c-Src variants; Figure S6: Analyses of cell proliferation and Myc, cyclin D1, and p27Kip1 in Sum159 and MDA-MB-231 cells expressing c-Src variants, considering—Doxy conditions as the control; Figure S7: Cell cycle analyses of SUM159 and MDA-MB-231 cells expressing c-Src variants; Figure S8: Migration and invasion data of SUM159 and MDA-MB-231 cells expressing c-Src variants; Figure S9: Kinetics curves of wound-healing assays of SUM159 and MDA-MB-231 cells expressing c-Src variants; Figure S10: Representative images of wound-healing assays of SUM159 and MDA-MB-231 cells expressing c-Src variants; Figure S11: Expression and activation of Caveolin 1, Paxillin, and Fak in SUM159 and MDA-MB-231 cells expressing c-Src variants. Referred to c-Src-wt as the control; Figure S12: Expression and activation of Caveolin 1, Paxillin, and Fak in SUM159 and MDA-MB-231 cells expressing c-Src variants. Referred to—Doxy as the control; Figure S13: Representative WB analyses of expression and activation of Caveolin 1, Paxillin, and Fak from the total cell extract from SUM159 and MDA-MB-231 cells expressing c-Src variants; Figure S14: Western Blot analyses of total c-Src, Fak, phosphorylated pY397-Fak, pY576-Fak, and Akt and phosphorylated pS473-Akt from SUM159 and MDA-MB-231 cells expressing c-Src variants; Figure S15: Confocal scanning microscopy analyses of pY14-Caveolin 1 and pY418-Src localization in SUM159 and MDA-MB-231 cells expressing c-Src variants; Figure S16: Kinetics curves of wound-healing assays of SUM159 and MDA-MB-231 cells treated with aptamers 14F and 17F; Figure S17: Representative images of wound-healing assays of SUM159 and MDA-MB-231 cells treated with aptamers 14F and 17F; Figure S18: Analyses by WB of expression of proliferation markers Myc, Cyclin D1, p27^Kip1^, and of migration Caveolin 1, Paxillin, and Fak in SUM159 and MDA-MB-231 cells treated with aptamers; Table S1: Detailed information for antibodies used in this work. Authentication of SUM159PT and MDA-MB-231 cell lines by short-tandem-repeat analyses. Uncropped gels of Figure 1, Figure 2, Figure 3 and Figure 4, S3, S7, S8, S9, S12, S13.
