# Supplementary material for: The Relevance of the SH2 Domain for c-Src Functionality in Triple-Negative Breast Cancer Cells
Source: Cancers (Basel). 2021 Jan 26;13(3):462. doi: 10.3390/cancers13030462 (PMC7865352; doi:10.3390/cancers13030462)
Supplement: Supplementary file 1 [file cancers-13-00462-s001.pdf]

The relevance of the SH2 domain for c-Src functionality in Triple-Negative Breast Cancer cells

Víctor Mayoral-Varo[1,*], Mª Pilar Sánchez-Bailon[1,2,*], Annarica Calcabrini[1,3,*], Marta García-Hernández[4], Valerio Frezza[4], Mª Elena Martín[4], Víctor M. González[4], Jorge Martín-Pérez[1,5, ‡]

Supplementary Materials

Supplementary Materials and methods

- **Purification and selection of aptamers for the SH2 domain of c-Src (Extended method)**
- **Immunofluorescence by lasers-scanning confocal microscopy**

Supplementary figures

- **Figure S1.** Analyses of Sphere Formation Efficiency (SFE).
- **Figure S2.** Images of mammospheres of SUM159 and MDA-MB-231 cells expressing c-Src variants.
- **Figure S3.** Effect of conditional expression of SrcDN in SUM159 and MDA-MB-231 or suppression of endogenous c-Src in MDA-MB-231 on SFE.
- **Figure S4.** Western Blots analyses of ALDH1, NANOG and Oct3-4 from mammospheres of SUM159 and MDA-MB-231 cells expressing c-Src variants.
- **Figure S5.** Soft-agar colonies from SUM159 and MDA-MB-231 cells expressing c-Src variants.
- **Figure S6.** Analyses of cell proliferation and Myc, cyclin D1, and p27Kip1 in Sum159 and MDA-MB-231 cells expressing c-Src variants, considering – Doxy conditions as Control.
- **Figure S7.** Cell cycle analyses of SUM159 and MDA-MB-231 cells expressing c-Src variants.
- **Figure S8.** Migration and invasion data of SUM159 and MDA-MB-231 cells expressing c-Src variants.
- **Figure S9.** Kinetics curves of wound-healing assays of SUM159 and MDA-MB-231 cells expressing c-Src variants.
- **Figure S10.** Representative images of wound-healing assays of SUM159 and MDA-MB-231 cells expressing c-Src variants.
- **Figure S11.** Expression and activation of Caveolin 1, Paxillin and Fak in SUM159 and MDA-MB-231 cells expressing c-Src variants. Referred to c-Src-wt as control.
- **Figure S12.** Expression and activation of Caveolin 1, Paxillin and Fak in SUM159 and MDA-MB-231 cells expressing c-Src variants. Referred to - Doxy as control.
- **Figure S13.** Representative WBs analyses of expression and activation of Caveolin 1, Paxillin and Fak from total cell extract from SUM159 and MDA-MB-231 cells expressing c-Src variants.
- **Figure S14.** Western Blot analyses of total c-Src, Fak, phosphorylated pY397-Fak, pY576-Fak, and Akt and phosphorylated pS473-Akt from SUM159 and MDA-MB-231 cells expressing c-Src variants.
- **Figure S15.** Confocal scanning microscopy analyses of pY14-Caveolin 1 and pY418-Src localization in SUM159 and MDA-MB-231 cells expressing c-Src variants.
- **Figure S16.** Kinetics curves of wound-healing assays of SUM159 and MDA-MB-231 cells treated with aptamers 14F and 17F.
- **Figure S17.** Representative images of wound-healing assays of SUM159 and MDA-MB-231 cells treated with aptamers 14F and 17F.
- **Figure S18.** Analyses by WB of expression of proliferation markers Myc, Cyclin D1, $p27^{Kip1}$ and of migration Caveolin 1, Paxillin and Fak in SUM159 and MDA-MB-231 treated with

aptamers.

- **Table S1.** Detail information for antibodies used in this work.
- **Authentication of SUM159PT and MDA-MB-231 cell lines by short-tandem-repeat analysis**
- **Uncropped gels of Figures 1, 2, 3, 4, S3, S7, S8, S9, S12, S13, S14.**

Supplementary Materials and methods

Purification and selection of aptamers for the SH2 domain of c-Src (Extended method)

1. Expression in E. coli and Purification of GST-SH2, GST-SH3 and GST-SH2-SH3

GST-SH2, GST-SH3 and GST-SH2-SH3 proteins containing human SH2 and SH3 human c-Src adapter domains were expressed, and soluble and insoluble fractions obtained. Both GST-SH2 and GST-SH3 were found in both insoluble and soluble fractions, while 100% of GST-SH2-SH3 was found in the insoluble fraction. GST-SH2 and GST-SH3 were purified from the soluble fraction by affinity chromatography with glutathione resin (Genescript).

2. Selection of a population of aptamers against GST-SH2.

Six rounds of selection were performed against GST-SH2, counteracting in round 3 and 5 with GST-SH3 following the next selection conditions: Selection buffer PBS + 1 mM Mg; temperature 37°C; incubation of the protein with the aptamer in solution and immobilization with glutathione resin.

The initial population (RND40) and rounds 3 and 6 were labelled with digoxigenin and the affinity of the populations for ELONA (Enzyme-Linked Oligonucleotide Assay) against GST-SH2 and GST-SH3 was analyzed.

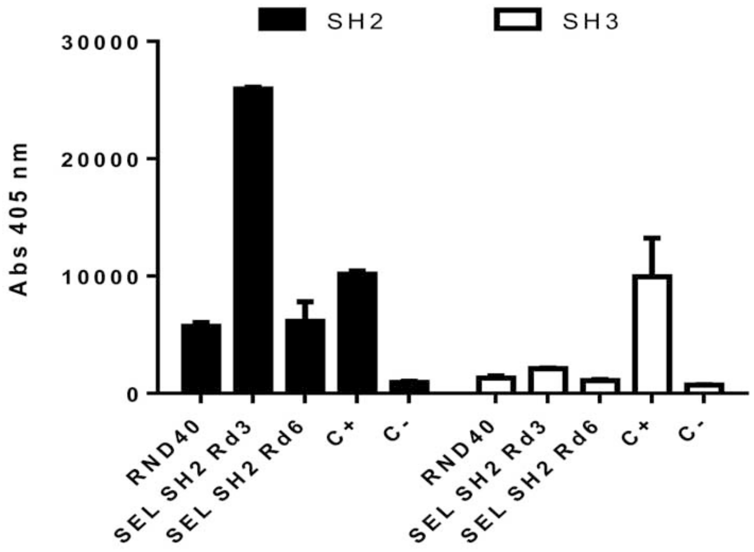

Figure 1Aptamers. The affinity of the populations against GST-SH2 and GST-SH3 was analyzed for ELONA. GST-SH2 and GST-SH3 proteins were plated in a 96-well plate. The initial population (RND40) and rounds 3 (SEL SH2 Rd3) and 6 (SEL SH2 Rd6) were labelled with digoxigenin and incubated in the presence of the proteins.

Data show that a significant enrichment was achieved in Round 3, however, it was lost in Round 6. None of the populations recognized GST-SH3. Round 3 (SEL SH2 Rd3) was cloned.

3. Cloning and characterization of individual aptamers against each peptide.

After cloning in the TA Cloning Kit (Invitrogen), 20 positive colonies were obtained. Plasmids were purified, individual aptamers amplified, and both strands were labelled with digoxigenin to check their affinity for ELONA.

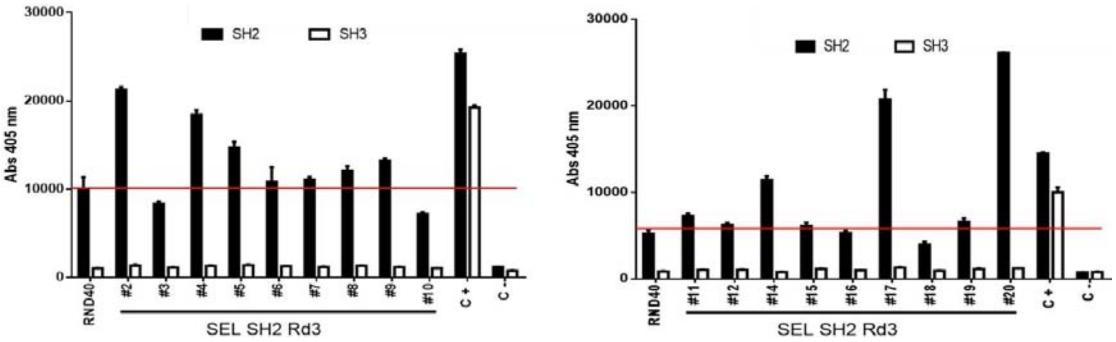

Figure 2 Aptamers. The affinity of the individual aptamers against GST-SH2 and GST-SH3 was analyzed for ELONA. GST-SH2 and GST-SH3 proteins were plated in a 96-well plate and the aptamers labelled with digoxigenin were incubated in the presence of the proteins.

In view of these results, clones 2, 4, 5, 14, 17 and 20 were selected and sequenced. In addition, the individual chains F3-digoxigenin and R3-digoxigenin were labelled to assess which chain displayed higher affinity.

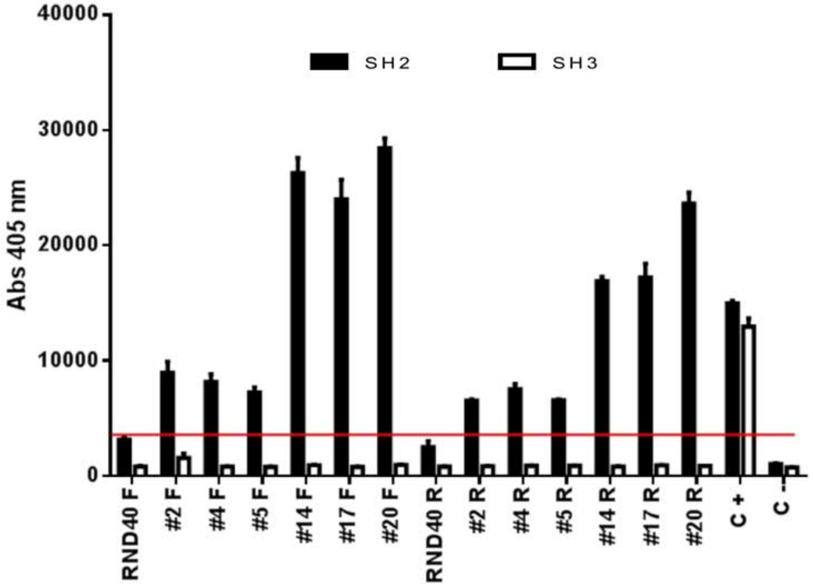

Figure 3 Aptamers. The affinity of the individual aptamers (F-chain and R-chain) against GST-SH2 and GST-SH3 was analyzed for ELONA. GST-SH2 and GST-SH3 proteins were plated in a 96-well plate and the aptamers labelled with digoxigenin were incubated in the presence of the proteins.

The results show that clones 14, 17 and 20 were the aptamers with the highest affinity, the binding capacity of the F chain being higher than that of R. Furthermore, the analysis of the sequences shows that aptamers 14 and 20 corresponded to the same sequence.

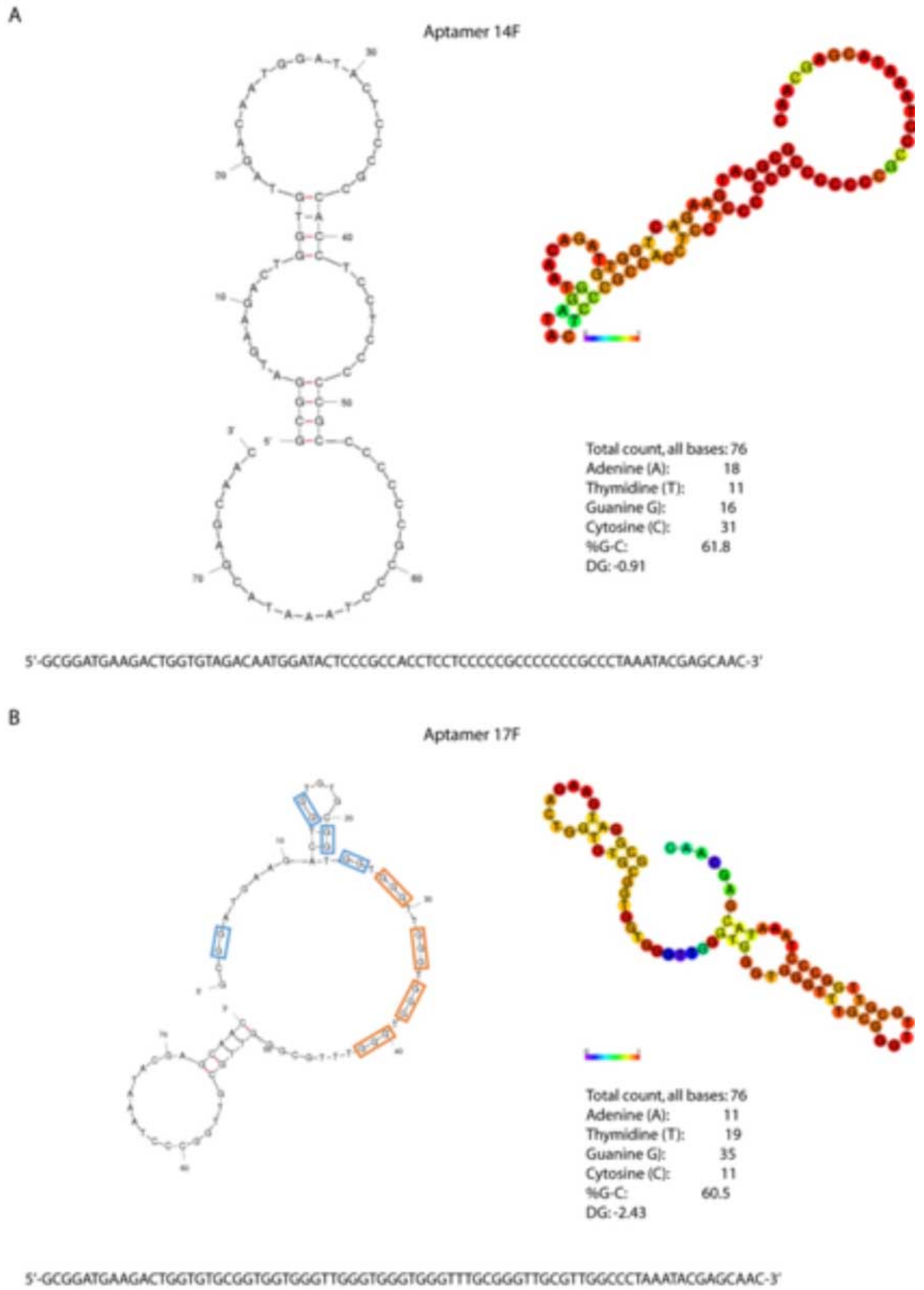

Figure 4 Aptamers. The structures obtained from in silico analysis of the aptamers ApSH2.14F and ApSH2.17F with the mFold and RNA fold programs as well as the presence of G-quadruplex analysed with QGRS mapper are shown.

Immunofluorescence by lasers-scanning confocal microscopy

Cells seeded on sterile coverslips were 72 h-treated with 0.2 μg/ml Doxy, fixed with 2% paraformaldehyde/PBS (15 min, room temperature), permeabilized with 0.5% Triton X100/PBS (10 min, room temperature) and blocked 1 h with 10% normal goat serum/PBS. Coverslips were incubated overnight at 4°C with primary antibodies diluted 1:50 in 5% normal goat serum/PBS: mouse monoclonal anti-pY14-Caveolin 1 and rabbit polyclonal anti-pY418-Src. Coverslips were washed with-PBS and then incubated for 1 h at 37°C with secondary-antibodies diluted 1:200 in 5% normal goat serum/PBS:–goat-anti-mouse IgG Alexa-Fluor 488 or goat-anti-rabbit IgG Alexa-Fluor 546 (Life Technologies). After washing with PBS, cells were counterstained with DAPI (D1306, Life Technologies) in PBS (10 μg/ml) for 5min at room temperature, washed in PBS and coverslips mounted on slides with ProLong reagent (Life Technologies). Samples were analyzed by confocal microscopy. Images were acquired using an inverted Zeiss LSM 710 laser-scanning microscope with a Plan Apochromat 60x/1.40 objective. Sequential scanning mode was used to avoid crosstalk between channels. Z-optical stacks with 0.6 μm intervals through the cell Z-axis were recorded. Images were processed with ZEN 2009 software (Carl Zeiss AG) and Adobe Photoshop CS5 (Adobe Systems In.).

Supplementary figures

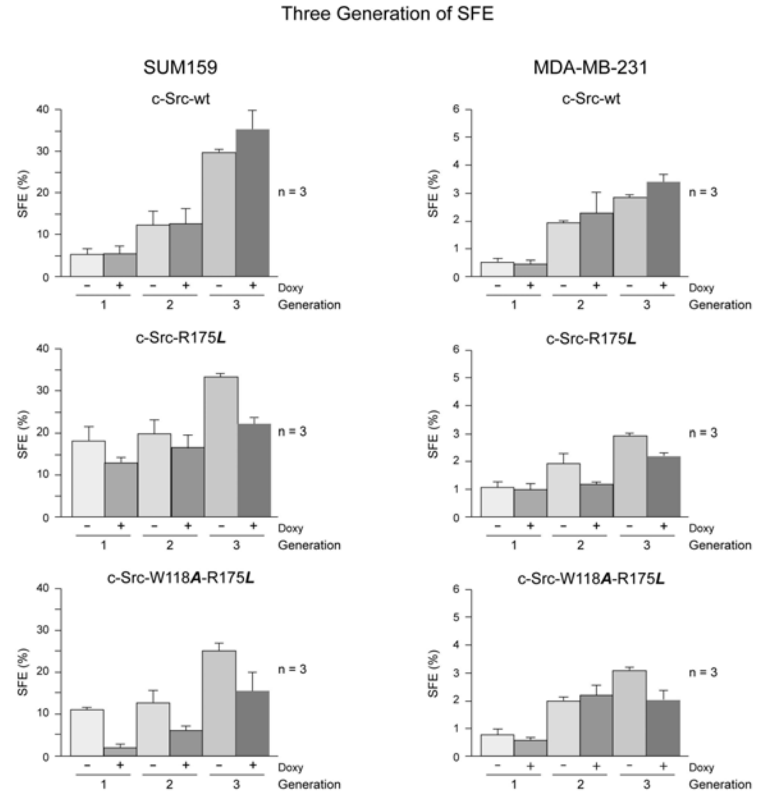

Figure S1. Analyses of three generations Sphere Formation Efficiency (SFE). The sphere formation ability was analyzed in three consecutive generations of SUM159 and MDA-MB-231 cells conditionally expressing chicken c-Src variants. Each experiment was measured in triplicates and repeated three times. Results were expressed as percentage of mean +/- SD.

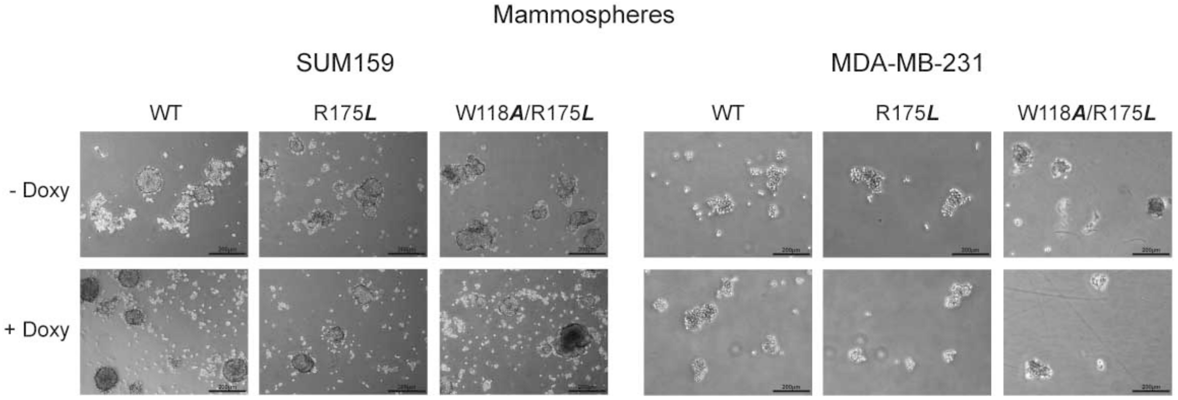

Figure S2. Representative images of mammospheres of SUM159 and MDA-MB-231 cells expressing c-Src variants.

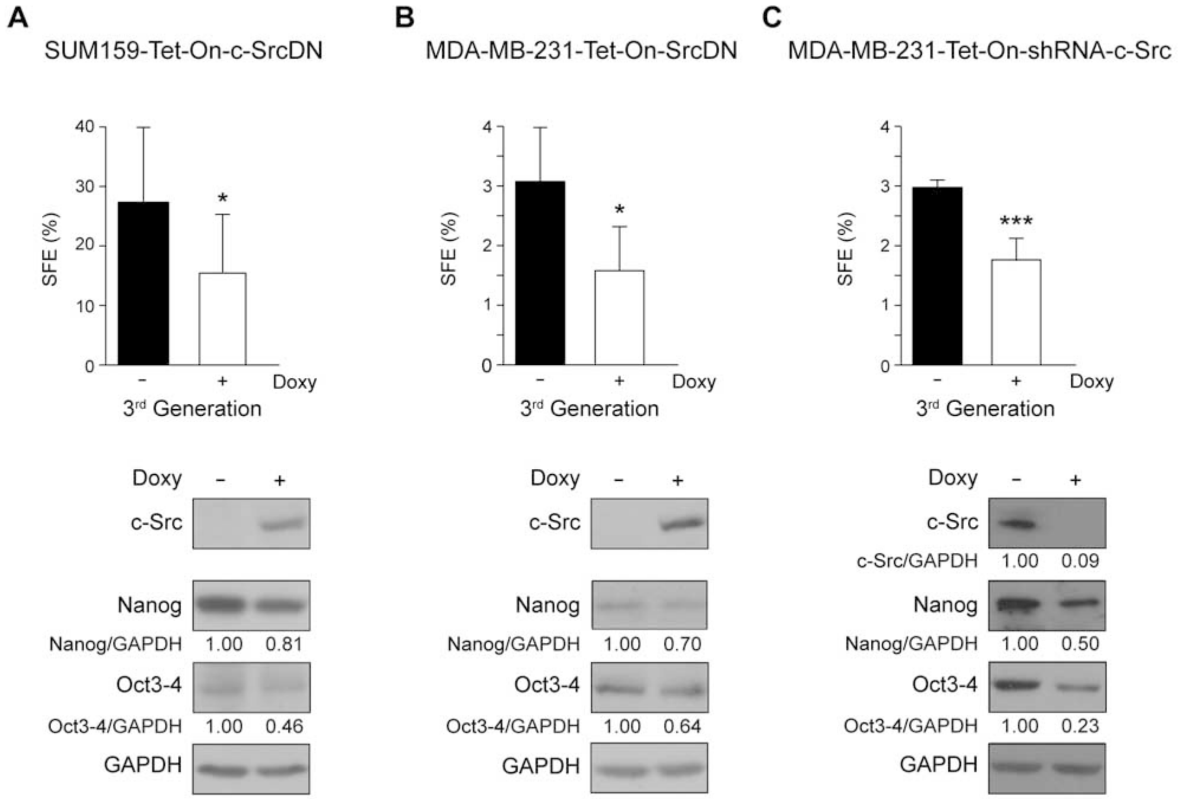

Figure S3. Effect of conditional expression of SrcDN in SUM159 and MDA-MB-231 or suppression of endogenous c-Src in MDA-MB-231 on SFE. (A) SUM159 and (**B**) MDA-MB-231 that conditionally expressed SrcDN (chicken c-Src K295M/Y527F) and (**C**) MDA-MB-231 with conditional suppression of endogenous c-Src were tested for SFE. Each experiment was measured in triplicates and repeated three times. Results of the third generation were expressed as percentage of mean +/- SD, p<0.5*, 0.01**, 0.001***. For each of these cell lines expression of c-Src, NANOG and Oct3/4 were determined by WB. GAPDH was analyzed for loading control.

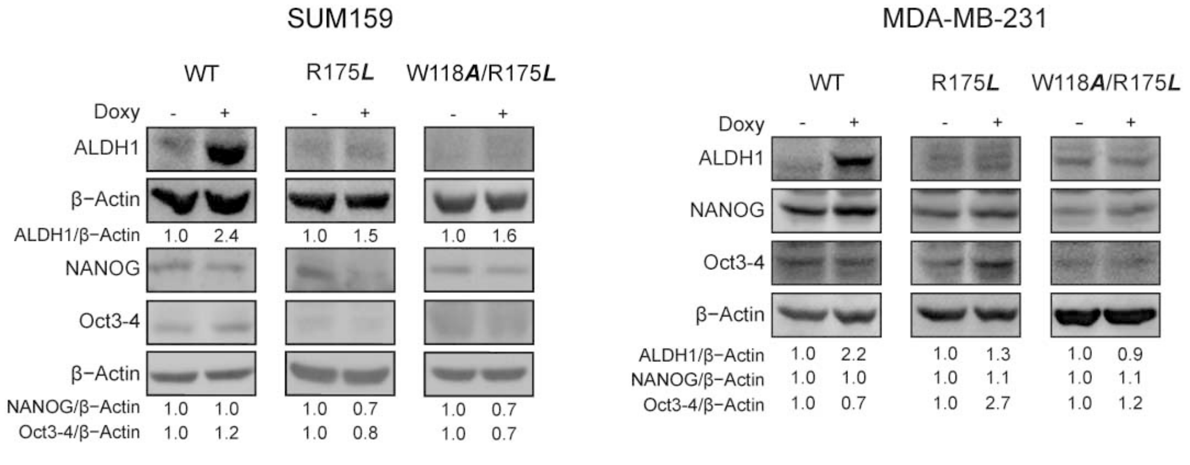

Figure S4. Representative Western Blots images and analyses of ALDH1, NANOG and Oct3-4 from mammospheres of SUM159 and MDA-MB-231 cells expressing c-Src variants.

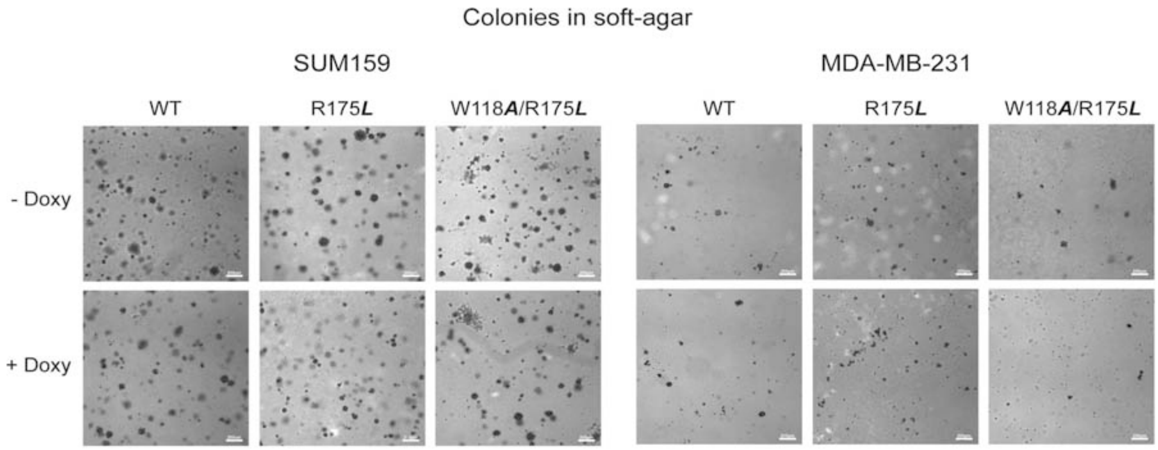

Figure S5. Soft-agar colonies from SUM159 and MDA-MB-231 cells expressing c-Src variants.

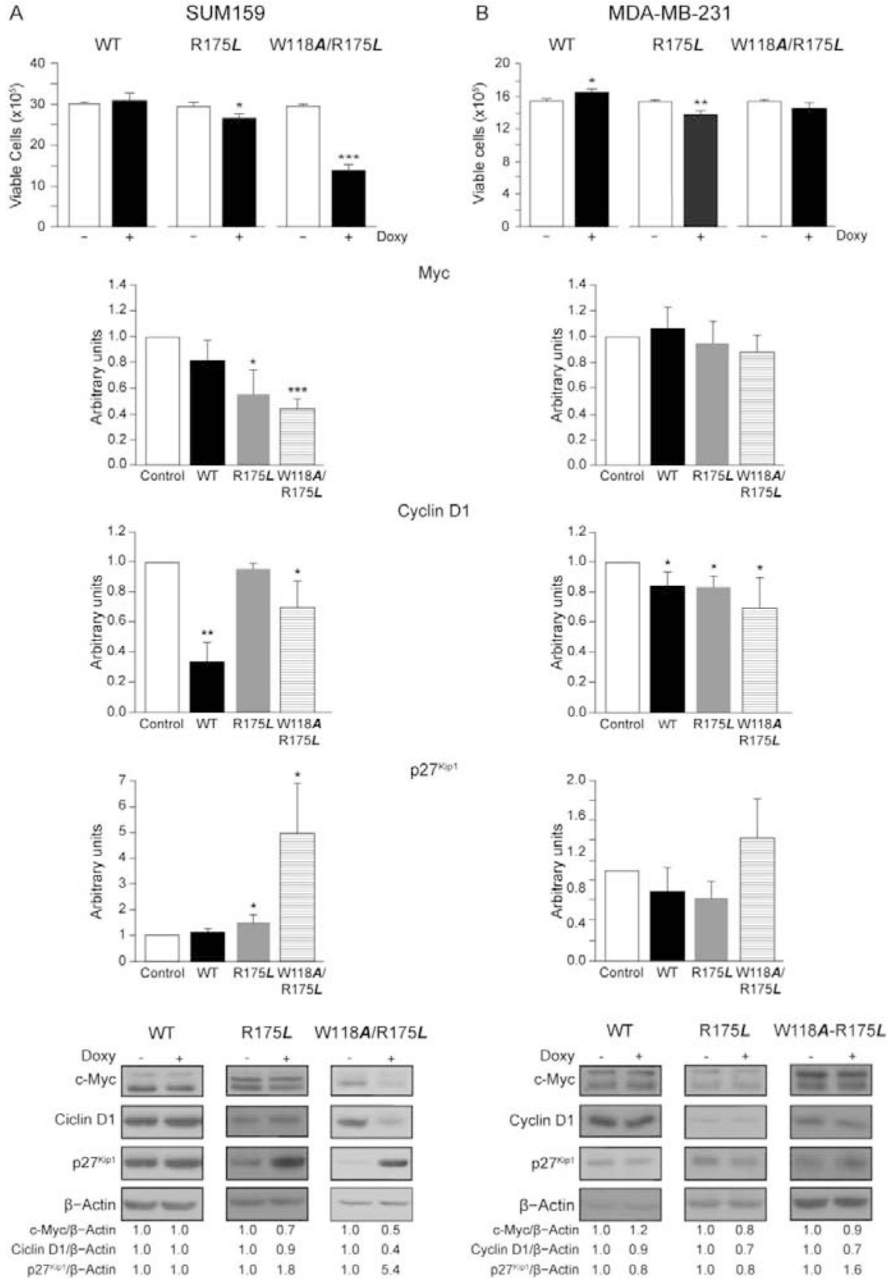

Figure S6. Analyses of cell proliferation and Myc, cyclin D1, and p27Kip1 in Sum159 and MDA-MB-231 cells expressing c-Src variants. Cells were cultured in absence (- Doxy = Control) or presence of Doxy (0.2 μg/mL) for 72h. Cells were then collected and analyzed as described in Material and methods. All the experiments were made in triplicates (N = 3). Results were expressed as mean +/- SD, p<0.5*, 0.01**, 0.001***.

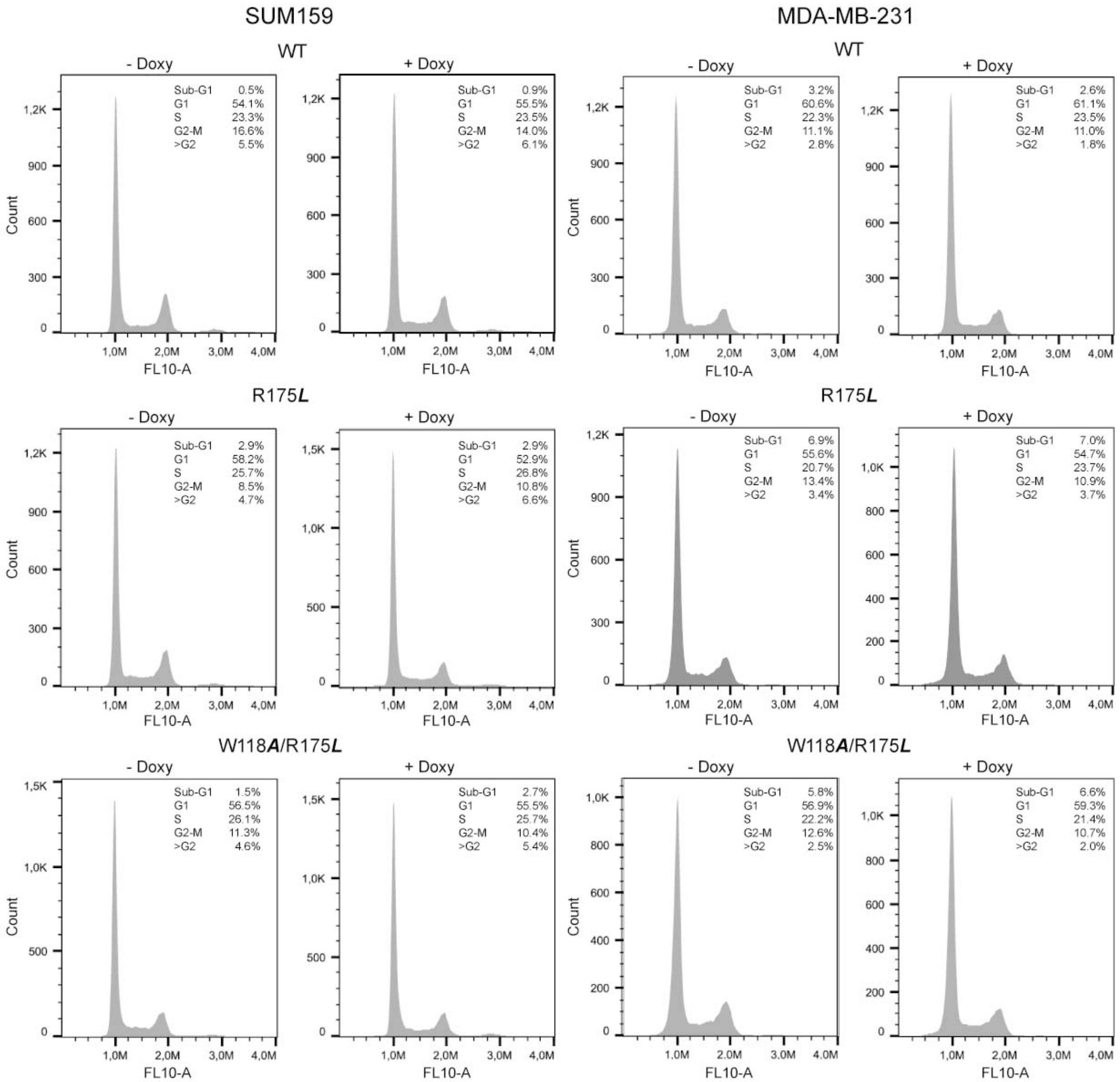

Figure S7. Cell cycle analyses of SUM159 and MDA-MB-231 cells expressing c-Src variants. SUM159 and MDA-MB-231 cultured for 72h in absence or presence of Doxy (0.2 μg/mL) were trypsinized, collected by centrifugation and the pellet resuspended in 70% EtOH overnight at 4ºC. Cells were then resuspended in PBS containing RNase and propidium iodide and incubated at 37ºC. The analyses were made in CytoFlex S (Beckman-Coulter) cytofluorimeter using the FlowJo software (Becton Dickinson), and the percentage of cells in Sub-G1, G1, S, G2-M and >G2 was calculated.

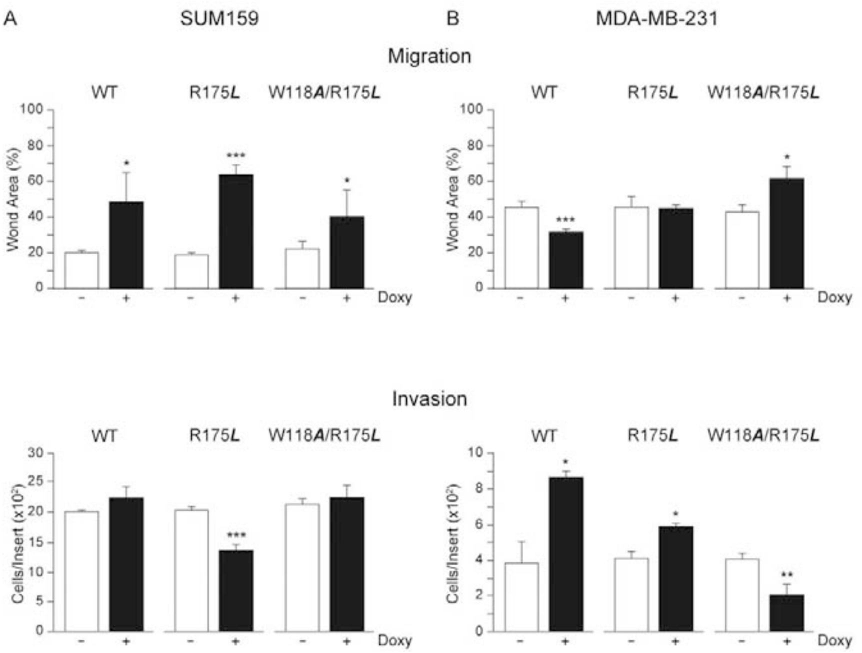

Figure S8. Migration and invasion data of SUM159 and MDA-MB-231 cells expressing c-Src variants. The quantitation of the assays was made in reference to cells untreated with Doxy as Control. All the experiments were made in triplicates (N = 3). Results of three independent experiments (N = 3) were expressed as mean +/- SD, p<0.5*, 0.01**, 0.001***.

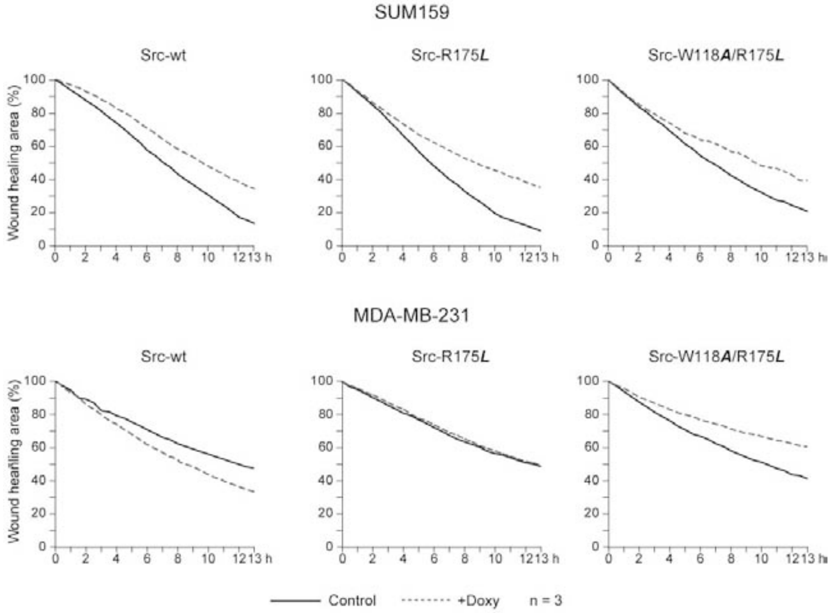

Figure S9. Kinetics curves of wound-healing assays of SUM159 and MDA-MB-231 cells expressing c-Src variants.

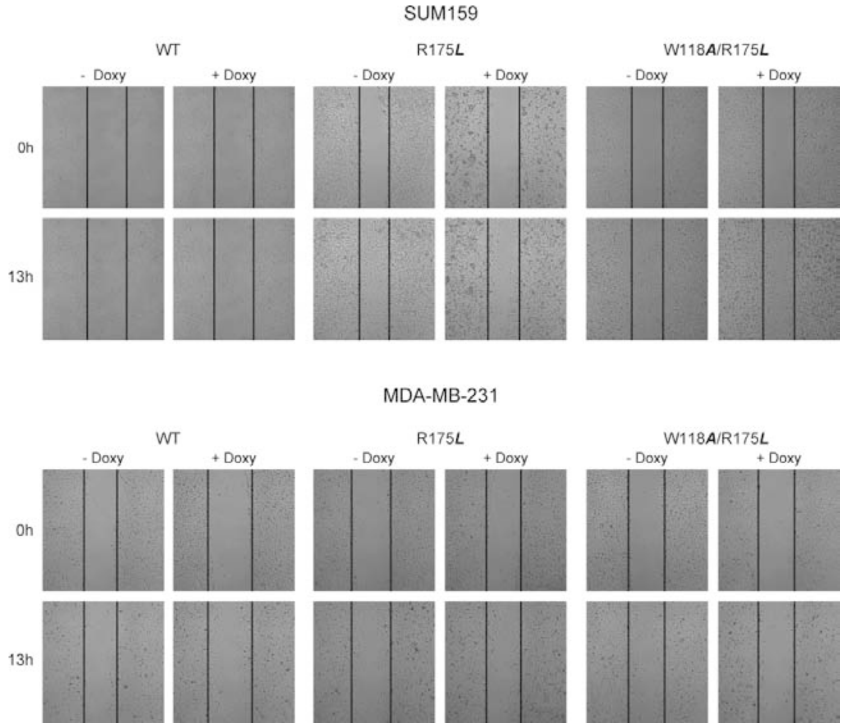

Figure S10. Representative images of wound-healing assays of SUM159 and MDA-MB-231 cells expressing c-Src variants.

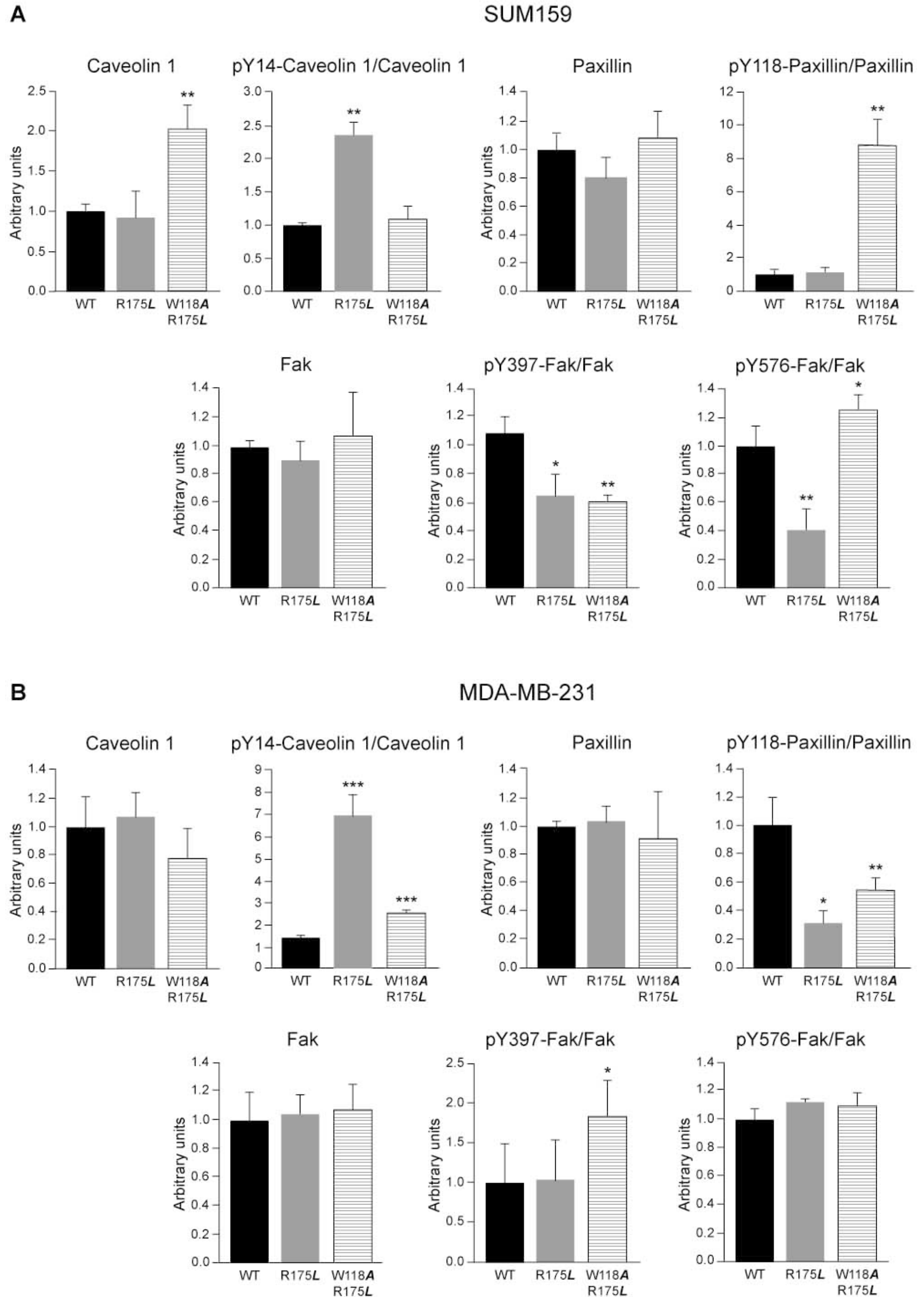

Figure S11. Expression and activation of Caveolin 1, Paxillin and Fak in SUM159 and MDA-MB-231. Expression and degree of activation of focal adhesion proteins Caveolin 1, Paxillin and Fak was determined by WB, scanned and expressed as arbitrary units in SUM159 (**A**) and MDA-MB-231 (**B**) that conditionally expressed c-Src variants. Results represented data obtained from three independent WB (N = 3) using either β-Actin, α-Tubulin or GAPDH as loading control expressed as percentage of mean +/- SD, and were referred to those obtained from cells expressing c-Src-wt considered as 1. The statistical significance is referred to cells expressing c-Src-wt, $p<0.5^*$, 0.01^{**}, 0.001^{***}.

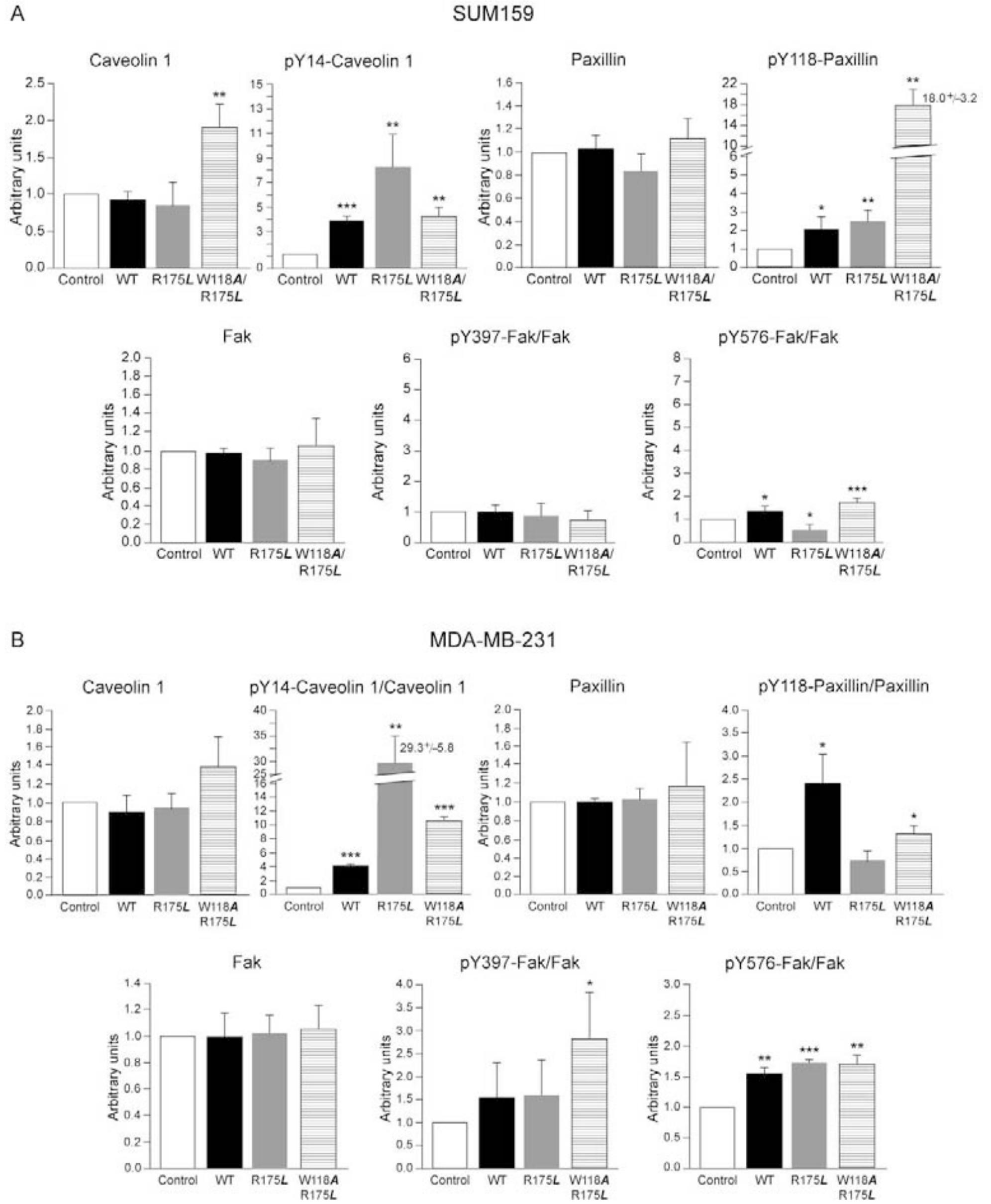

Figure S12. Expression and activation of Caveolin 1, Paxillin and Fak in SUM159 and MDA-MB-231. Expression and degree of activation of focal adhesion proteins Caveolin 1, Paxillin and Fak was determined by WB, scanned and expressed as arbitrary units in SUM159 (**A**) and MDA-MB-231 (**B**) that conditionally expressed c-Src variants. Results represented data obtained from three independent WB (N = 3) using either β-Actin, α-Tubulin or GAPDH as loading control expressed as percentage of mean +/- SD, and were referred to those obtained from cells untreated with Doxy (- Doxy = Control) considered as 1. The statistical significance is referred to cells expressing c-Src-wt, p<0.5*, 0.01**, 0.001***.

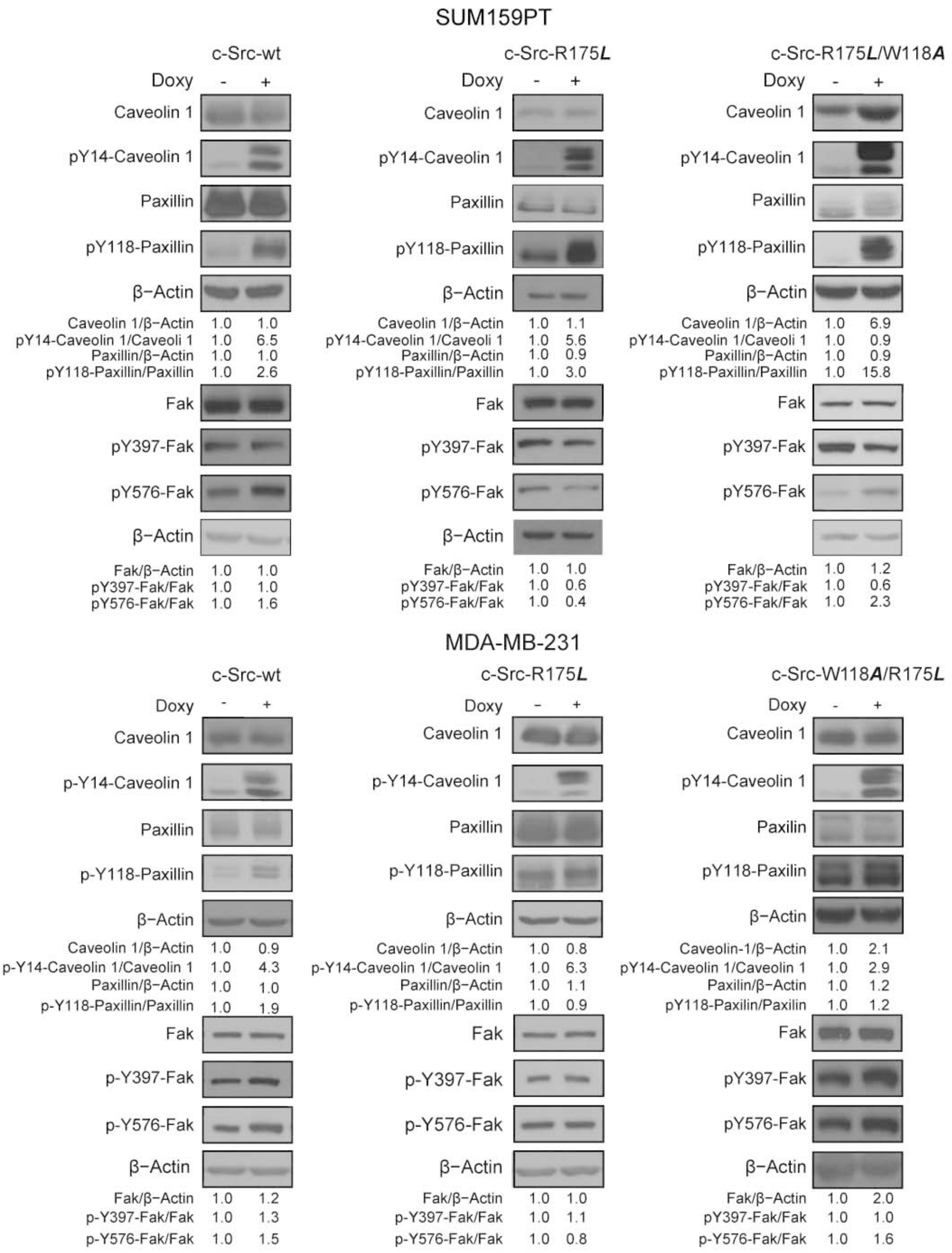

Figure S13. Representative WBs analyses of expression and activation of Caveolin 1, Paxillin and Fak from total cell extract from SUM159 and MDA-MB-231 cells expressing c-Src variants.

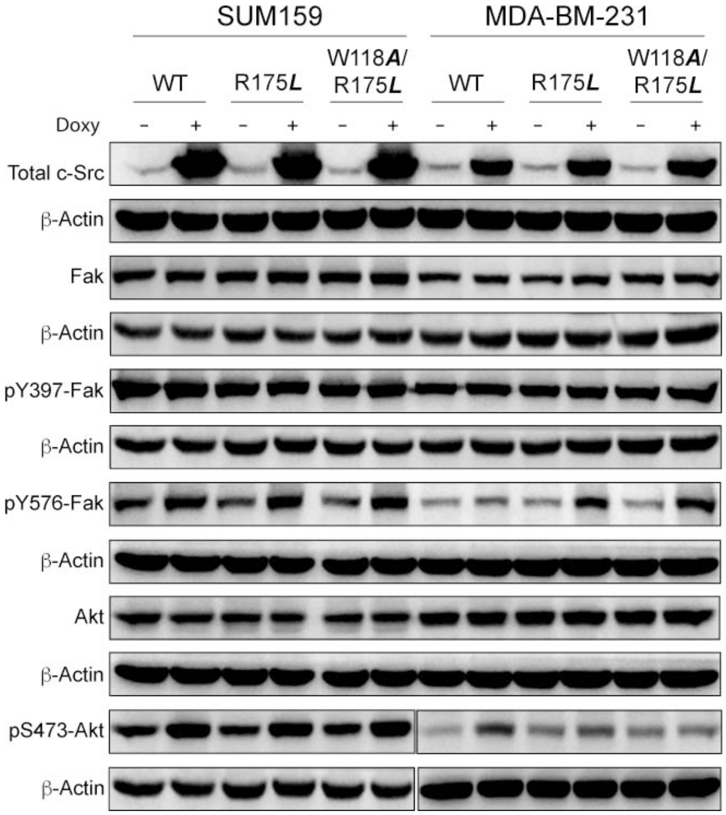

Figure S14. Western Blot analyses of total c-Src, Fak, phosphorylated pY397-Fak, pY576-Fak, and Akt and phosphorylated pS473-Akt from SUM159 and MDA-MB-231 cells expressing c-Src variants. Total cell extracts were prepared and WB analyses were made as described in Materials and methods. Since the intensity of the signals from WBs for pS473-Akt in SUM159 cell extracts were much more intense than those in MDA-MB-231, we reblotted this part of the membrane again with pS473-Akt antibody and with its corresponding β-Actin for loading control.

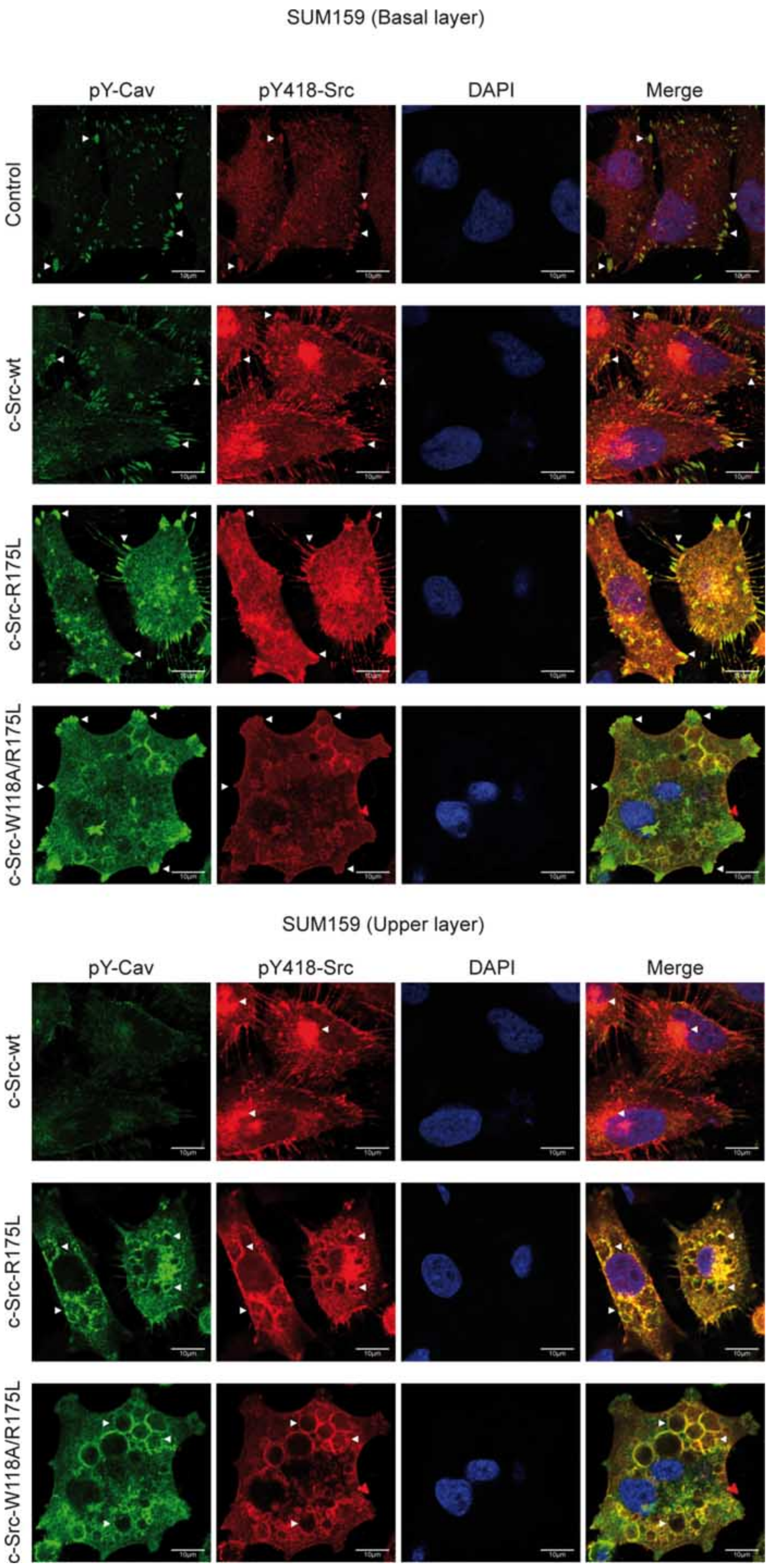
SUM159 (Basal layer)
pY-Cav
pY418-Src
DAPI
Merge
Control
c-Src-wt
c-Src-R175L
c-Src-W118A/R175L
SUM159 (Upper layer)
pY-Cav
pY418-Src
DAPI
Merge
c-Src-wt
c-Src-R175L
c-Src-W118A/R175L

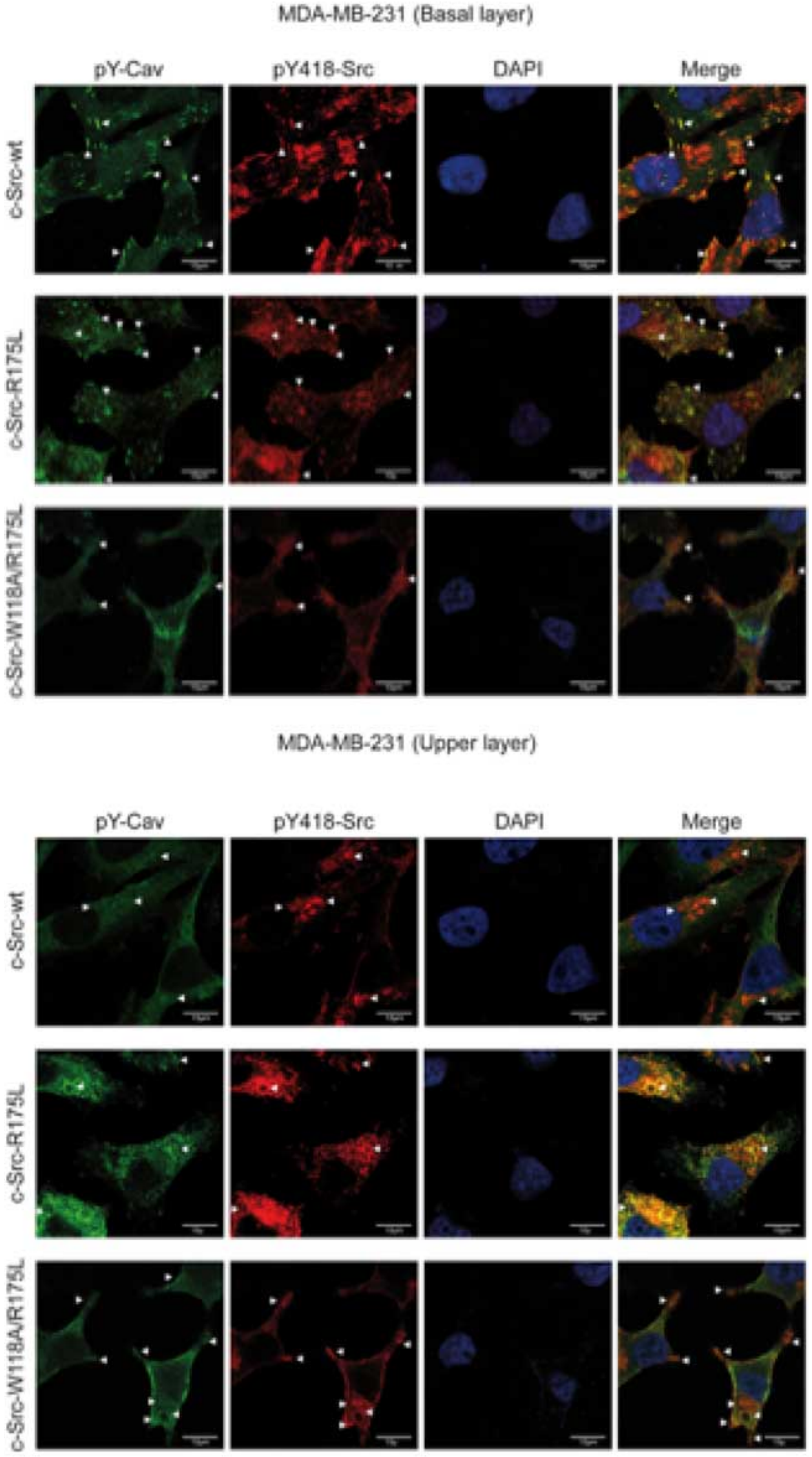

Figure S15. Confocal scanning microscopy analyses of pY14-Caveolin 1 and pY418-Src localization in SUM159 and MDA-MB-231 cells. Control, c-Src expressing variants SUM159 and MDA-MB-231 were seeded in coverslips and then incubated with anti-pY14-Caveolin 1, anti-pY418-Src and DAPI

as described in Supplementary methods and analyzed by scanning confocal microscopy at two levels, Basal layer for SUM159 and MDA-MB-231 cells respectively, and Upper layer for SUM159 and MDA-MB-231 cells respectively. White arrows showed localization of the antigens. Bar = 10 μm.

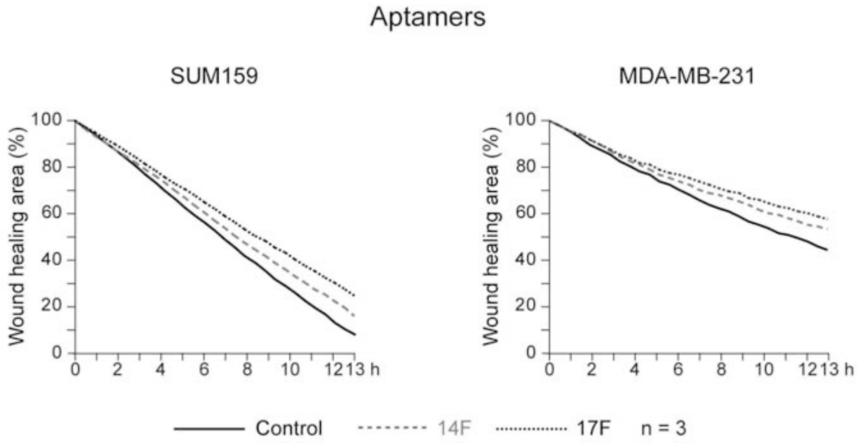

Figure S16. Kinetics curves of wound-healing assays of SUM159 and MDA-MB-231 cells. treated with aptamers 14F and 17F.

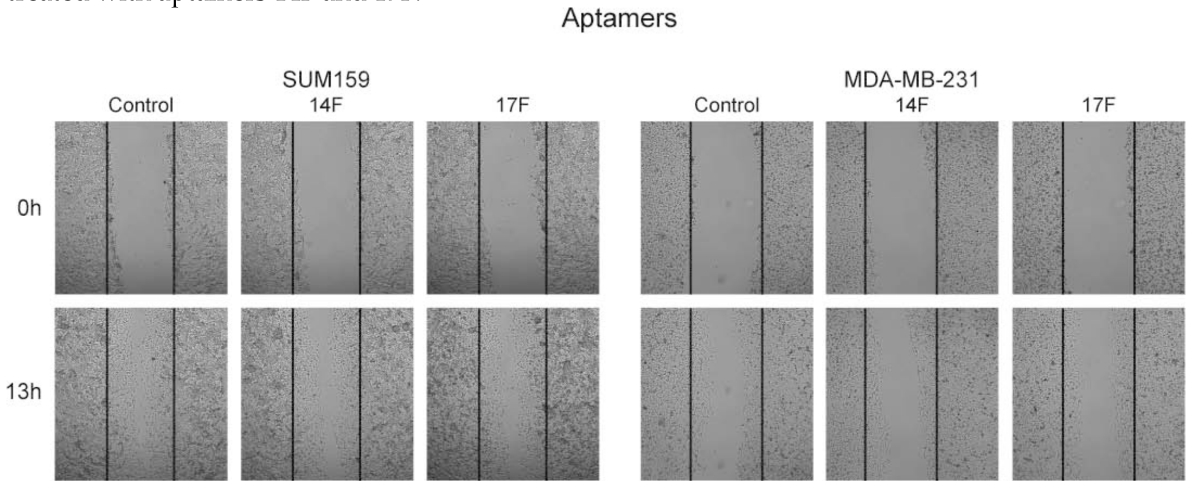

Figure S17. Representative images of wound-healing assays of SUM159 and MDA-MB-231 cells treated with aptamers 14F and 17F.

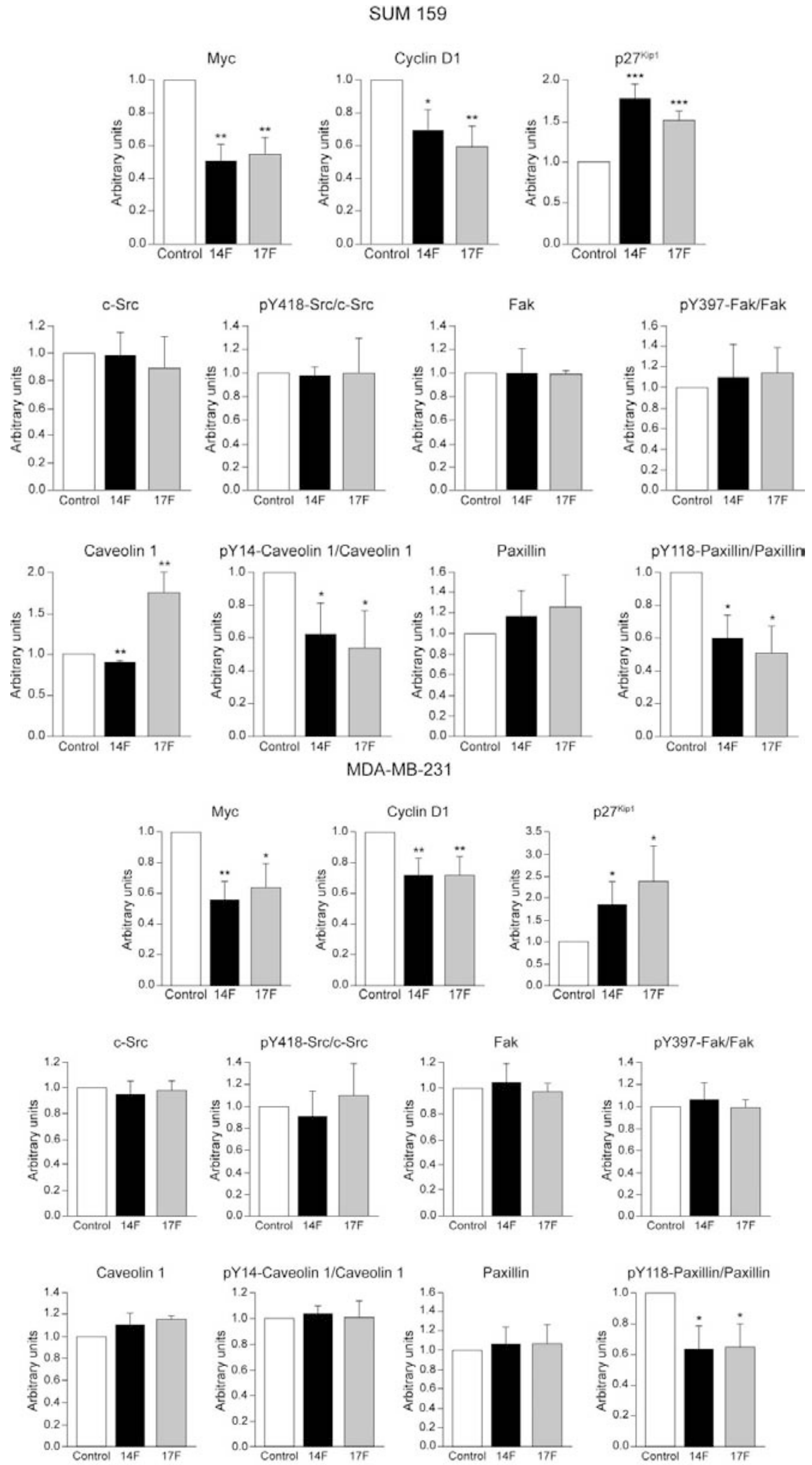

Figure S18. Analyses by WB of expression of proliferation markers Myc, Cyclin D1, p27^{Kip1} and of migration Caveolin 1, Paxillin and Fak in SUM159 and MDA-MB-231 treated with aptamers.

Table S1. Detailed information for antibodies used in this work.

Name	Type	Supplier	Catalog #	Origin	Dilution Ratio WB*/IF**
β-Actin	mouse monoclonal	Sigma-Aldrich (Merck)	A5441	San Luis, MI, USA	1:1000/…
ALDH1	mouse monoclonal	BD-Biosciences	#611194	Franklin Lakes, NJ, USA	1:500/…
Akt	mouse monoclonal	Cell Signaling	#2920	Danvers, MA, USA	1:1000/…
Akt-pS473	rabbit polyclonal	Cell Signaling	#4060	Danvers, MA, USA	1:1000/…
Caveolin 1	rabbit polyclonal	BD-Biosciences	#610059	Franklin Lakes, NJ, USA	1:5000/…
Caveolin 1-pY14	mouse monoclonal	BD-Biosciences	#611338	Franklin Lakes, NJ, USA	1:1000/1:50
Cyclin D1 (H-295)	rabbit polyclonal	Santa Cruz Biotechnology, Inc.	sc-753	Dallas, TX, USA	1:1000/…
c-Src (MAb-327)	mouse monoclonal	J.S. Brugge (Harvard University)		Harvard University, MA, USA	1:1000/…
c-Src (MAb EC10)	mouse monoclonal	Millipore (Merk)	#05-185	Billerica, MA, USA	1:1000/…
c-Src-pY418	rabbit polyclonal	Invitrogen	#44660G	Camarillo, CA, USA	1:1000/1:50
Fak	rabbit polyclonal	Santa Cruz Biotechnology, Inc.	Sc-557	Dallas, TX, USA	1:1000/…
Fak-pY397	mouse monoclonal	BD-Biosciences	#611722	Franklin Lakes, NJ, USA	1:1000/…
Fak-pY576	rabbit polyclonal	Invitrogen	#44652	Camarillo, CA, USA	1:1000/…
Fyn	rabbit polyclonal	Santa Cruz Biotechnology, Inc.	sc-16	Dallas, TX, USA	1:1000/…
GAPDH (clone 6C5)	mouse monoclonal	Millipore (Merk)	CB1001	Billerica, MA, USA	1:4000/…
Lyn	rabbit polyclonal	Santa Cruz Biotechnology, Inc.	sc-7274	Dallas, TX, USA	1:1000/…
Myc	rabbit polyclonal	Santa Cruz Biotechnology, Inc.	sc-764	Dallas, TX, USA	1:1000/…
Nanog	rabbit polyclonal	Millipore (Merk)	#AB9220	Billerica, MA, USA	1:1000/…
Oct3/4	goat polyclonal	Santa Cruz Biotechnology, Inc.	Sc-8629	Dallas, TX, USA	1:500/…
Paxillin	mouse monoclonal	BD-Biosciences	#610051	Franklin Lakes, NJ, USA	1:1000/…
Paxillin-pY118	rabbit polyclonal	Cell Signaling	#2541	Danvers, MA, USA	1:1000/…
p27	mouse monoclonal	Invitrogene	AHZ0452	Camarillo, CA, USA	1:1000/…
α-Tubulin	mouse monoclonal	Sigma-Aldrich (Merck)	T9026	San Luis, MI, USA	1:8000/…
Yes	mouse monoclonal	Santa Cruz Biotechnology, Inc.	sc-48396	Dallas, TX, USA	1:1000/…
Goat-anti-Mouse-Alexa-Fluor 488	goat polyclonal	Invitrogen	#A32723	Camarillo, CA, USA	……./1:200
Goat-anti-Rabbit-Alexa-Fluor 546	goat polyclonal	Invitrogen	#A-11030	Camarillo, CA, USA	……./1:200
Goat-anti-Mouse-HRP	goat polyclonal	Santa Cruz Biotechnology, Inc.	sc-2005	Dallas, TX, USA	1:3000/…
Goat-anti-Rabbit-HRP	goat polyclonal	Invitrogen	#21234	Camarillo, CA, USA	1:5000/…

Western Blotting (WB)*
Immunofluorescence (IF)**

Table S1. Detail information for antibodies used in this work

HUMAN CELL LINE STR PROFILE REPORT

CELL LINE DESCRIPTION according to customer: SUM-159PT (CVCL-5423)

Sample Submitted By: Jorge Martín Pérez
Instituto de Investigaciones Biomédicas. CSIC
jmartin@iib.uam.es

Date: 16.01.17
Microsatellite Run Number (pdf attached): M131
Laboratory: Genomics Core Facility.
Instituto de Investigaciones Biomédicas "Alberto Sols" CSIC-UAM.
28029 Madrid
00 34 91 585 44 74 (Tel.) 00 34 91 585 44 01 (Fax)
genomica@iib.uam.es

STR PROFILE DATA:

STR PROFILE OBTAINED		EXPECTED
D5S818	11	11
D13S317	12	12
D7S820	10	10
D16S539	11	11
vWA	16	16
TH01	6.7	6.7
TPOX	8,11	8,11
CSF1PO	10,11	11
D21S11	28	28
Amelogenin	X	X
RESULTS		
Number of shared alleles: 16		
Number of alleles in database: 16		
Percent match: 100%		

TEST RESULTS:
Query profiles were compared with data supplied by researcher
Cell lines with ≥80% match are considered to be related (i.e common ancestor).
Cell lines with 55%-80% match require further profiling

- ✓ The submitted sample profile is human and matches 100% with customer cell line description
- The submitted sample profile is human, but does not match customer cell line description
- No human STRs are amplified

DATABASE OUTPUT:

See: http://web.expasy.org/cellosaurus/CVCL_5423

METHOD:

STR ANALYSIS	
STR amplification kit	GenePrint^R 10 System (Promega)
STR profile analysis software	GeneMapper® v3.7 (Life Technologies)
Genomic Analyzer System	ABI 3130 XL (Applied Biosystems)
DNA source	Cultured cells; cultured cells pellet
DNA isolation method	DNeasy blood and tissue kit (Qiagen)
DNA quantification method	Qubit 2.0 Fluorometer (Life Technologies)
Amount of DNA/amplification	4 ng

The *GenePrint*® 10 System allows co-amplification and three-color detection of ten human loci: TH01, TPOX, vWA, Amelogenin, CSF1PO, D16S539, D7S820, D13S317, D21S11 and D5S818. These loci collectively provide a genetic profile with a random match probability of 1 in 2.92 × 10^9 and are used for human cell line and tissue authentication and identification and human cell line cross-contamination determination. Samples are processed using the ABI Prism® 31300xl Genetic Analyzer. Data are analyzed using GeneMapper® ID-X v1.2 software (Applied Biosystems). STRs profiles are sent for comparison against cell line date bases like ATCC (American Type Culture Collection), DSMZ (Deutsche Sammlung von Mikrorganismen and Zellkulturen)...

HUMAN CELL LINE STR PROFILE REPORT

ADDITIONAL INFORMATION RELATED TO CELL LINE SUM-159PT ACCORDING TO THE FOLLOWING REFERENCE

REFERENCE: A resource for cell line authentication, annotation and quality control. Nature 520, 307. 2015

ANNOTATIONS (Supplementary Table 2):

Cell Line Name	Canonical Name	Species	Primary Tissue	Site of Extraction	Tissue Diagnosis	Gender	Ethnicity	Age	Age Unit	Age Category	SOURCE	SOURCE	Synonym Group	NOTES
SUM 159PT	SUM 159PT	Homo sapiens	Breast		Anaplastic Carcinoma	Female	Unknown	Unknown	Unknown	Unknown	Asterand	Asterand	--	--

SYNONYMOUS CELL LINES (Supplementary Table 6): None

MISREPORTED SYNONYMOUS CELL LINES (Supplementary Table 7): No

ELECTROPHEROGRAM

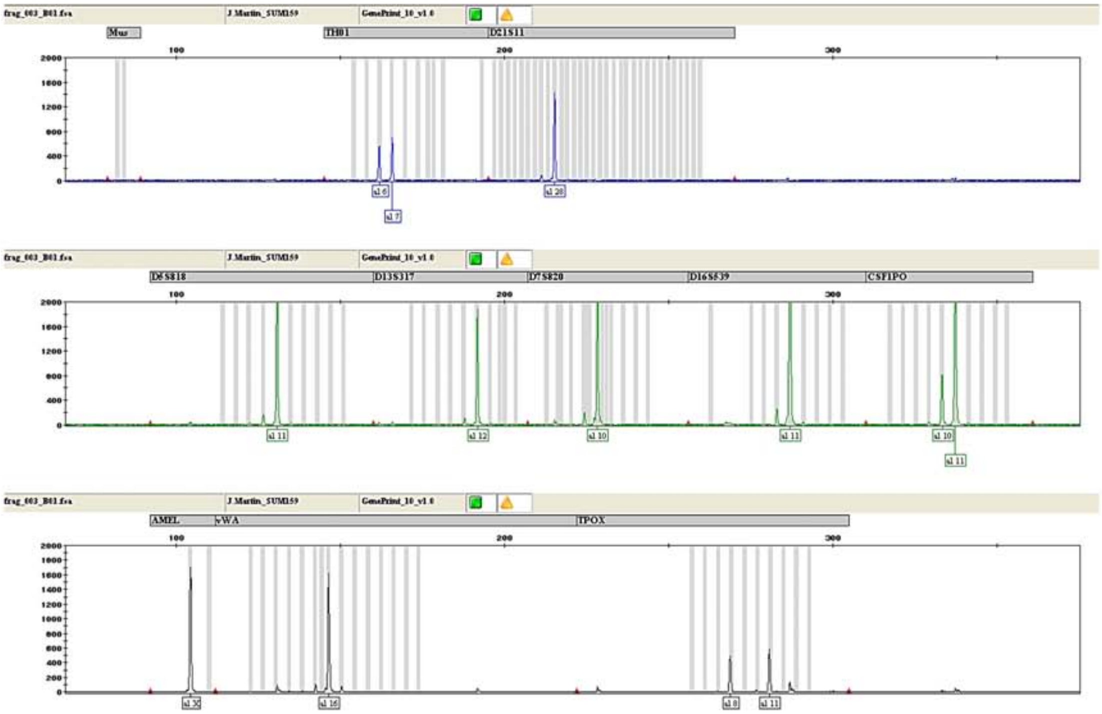

HUMAN CELL LINE STR PROFILE REPORT

CELL LINE DESCRIPTION according to customer: MDA-MB-231 (2) (ATCC ® HTB-26)

Sample Submitted By: Jorge Martín Pérez
Instituto de Investigaciones Biomédicas. CSIC
jmartin@iib.uam.es

Date: 13.12.2018
Microsatellite Run Number (pdf attached): M192
Laboratory: Genomics Core Facility.
Instituto de Investigaciones Biomédicas "Alberto Sols" CSIC-UAM.
28029 Madrid
00 34 91 585 44 74 (Tel.) 00 34 91 585 44 01 (Fax)
genomica@iib.uam.es

STR PROFILE DATA:

STR PROFILE OBTAINED		EXPECTED
D5S818	12	12
D13S317	13	13
D7S820	8,9	8,9
D16S539	12	12
vWA	15,18	15,18
TH01	7,9.3	7,9.3
TPOX	8,9	8,9
CSF1PO	12,13	12,13
D21S11	33.2	
Amelogenin	X	X
RESULTS		
Number of shared alleles: 16		
Number of alleles in database: 16		
Percent match: 100%		

TEST RESULTS:
Query profiles were sent against ATCC database
Cell lines with ≥80% match are considered to be related (i.e common ancestor).
Cell lines with 55%-80% match require further profiling

- ✓ The submitted sample profile is human and matches 100% with customer cell line description
- The submitted sample profile is human, but does not match customer cell line description
- No human STRs are amplified

DATABASE OUTPUT:

%Match	ATCC® Number	Designation	D5S818	D13S317	D7S820	D16S539	vWA	TH01	AMEL	TPOX	CSF1PO
100.0	HTB-26	MDA-MB-231Breast AdenocarcinomaHuman	12	13	8,9	12	15,18	7,9.3	X	8,9	12,13

METHOD:

STR ANALYSIS	
STR amplification kit	GenePrint^R 10 System (Promega)
STR profile analysis software	GeneMapper® v3.7 (Life Technologies)
Genomic Analyzer System	ABI 3130 XL (Applied Biosystems)
DNA source	Cultured cells; cultured cells pellet
DNA isolation method	DNeasy blood and tissue kit (Qiagen)
DNA quantification method	Qubit 2.0 Fluorometer (Life Technologies)
Amount of DNA/amplification	4 ng

The *GenePrint*® 10 System allows co-amplification and three-color detection of ten human loci: TH01, TPOX, vWA, Amelogenin, CSF1PO, D16S539, D7S820, D13S317, D21S11 and D5S818. These loci collectively provide a genetic profile with a random match probability of 1 in 2.92 × 10^9 and are used for human cell line and tissue authentication and identification and human cell line cross-contamination determination. Samples are processed using the ABI Prism® 31300xl Genetic Analyzer. Data are analyzed using GeneMapper® ID-X v1.2 software (Applied Biosystems). STRs profiles are sent for comparison against cell line date bases like ATCC (American Type Culture Collection), DSMZ (Deutsche Sammlung von Mikrorganismen and Zellkulturen)...

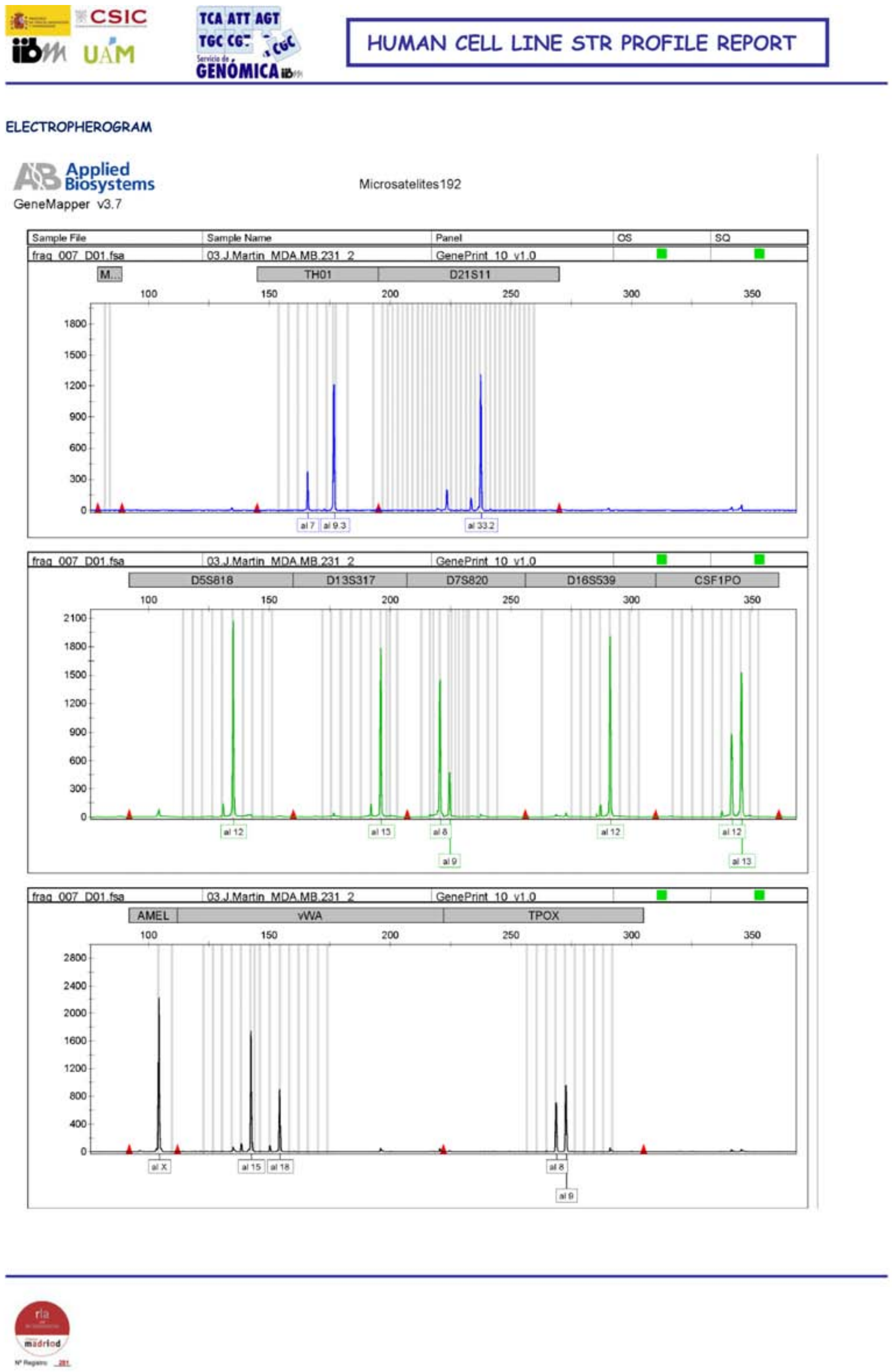

Authentication of SUM159PT and MDA-MB-231 cell lines by short-tandem-repeat analysis

(GenePrintR 10 System from Promega, and GeneMapper v3.7 STR profile analysis software, Life Technologies)

Figure 1_B

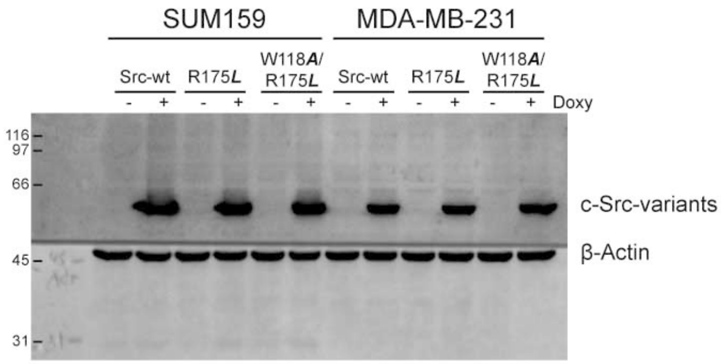

Figure 1_C

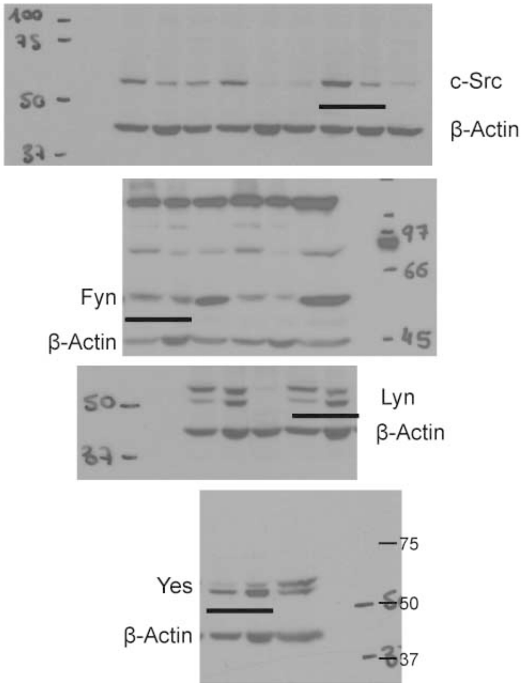

Uncropped gels corresponding to Fig 1 B, C

Western Blots for Mammospheres of SUM159

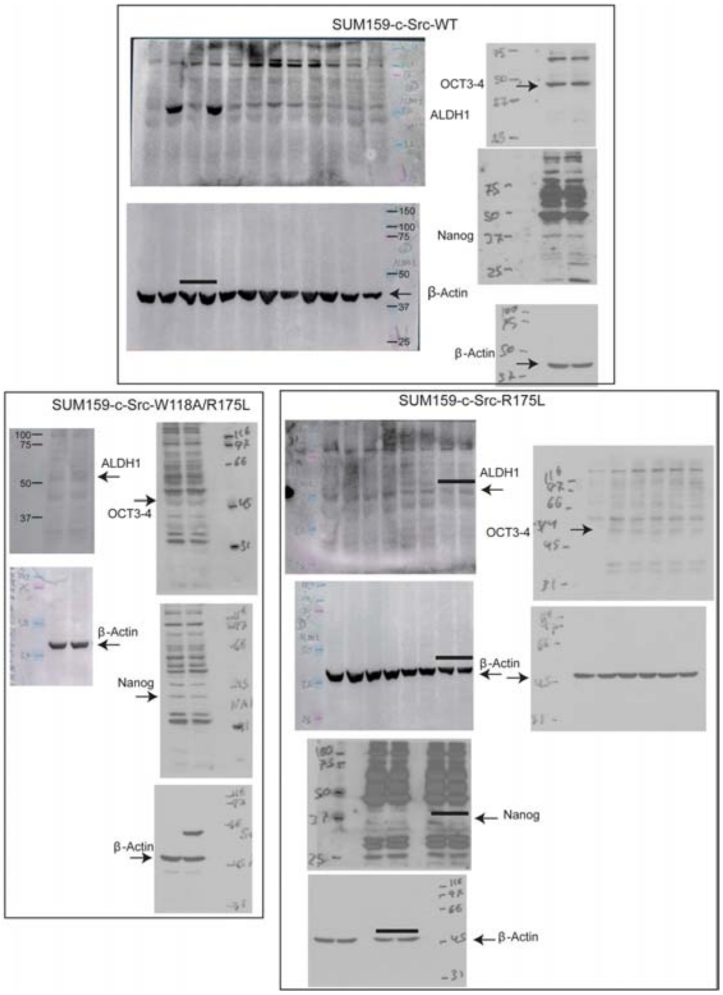

Uncropped gels corresponding to Fig. 2 C and Fig. S3

Western Blots for Mammospheres of MDA-MB-231

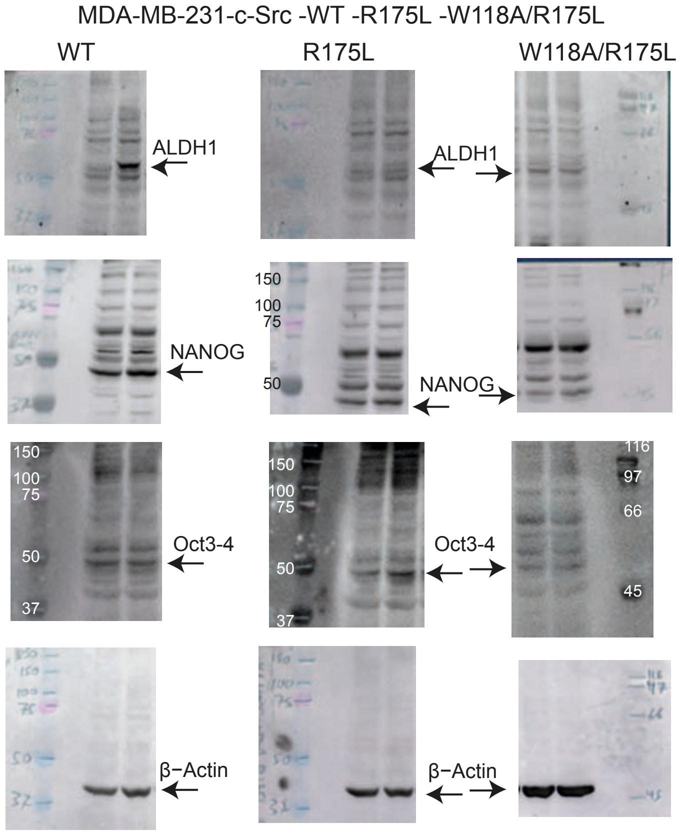

Uncropped gels corresponding to Fig. 2 D and Fig. S3

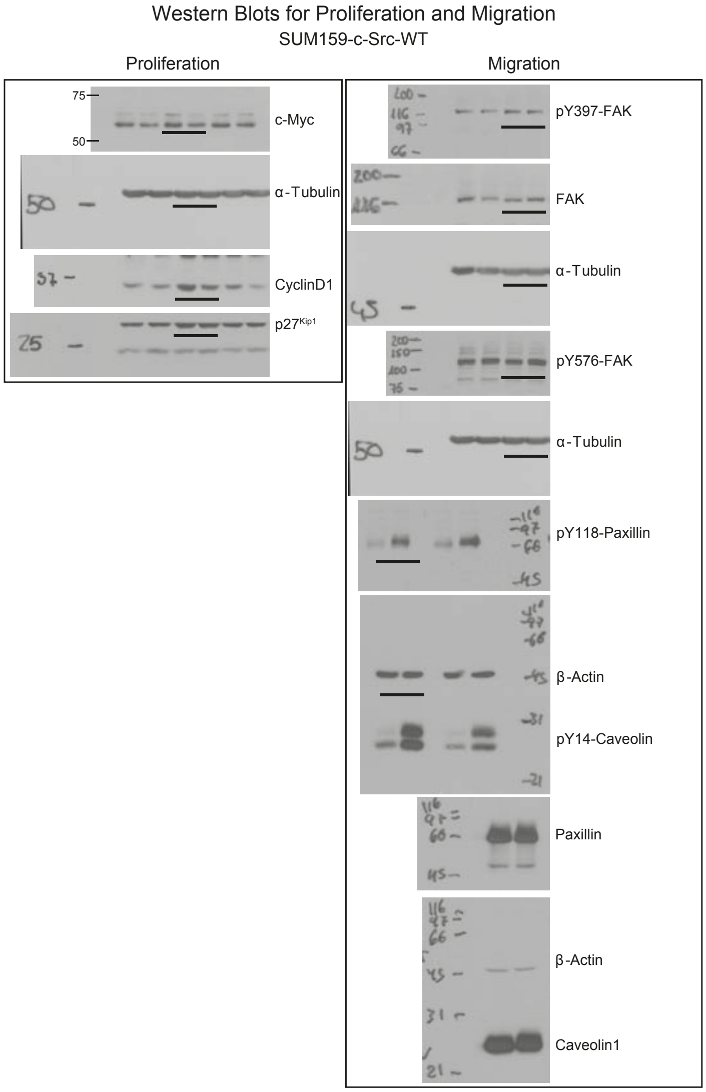

Uncropped gels corresponding to Figures 3, S7, 4, S9, S12 and S13.

Western Blots for Proliferation and Migration

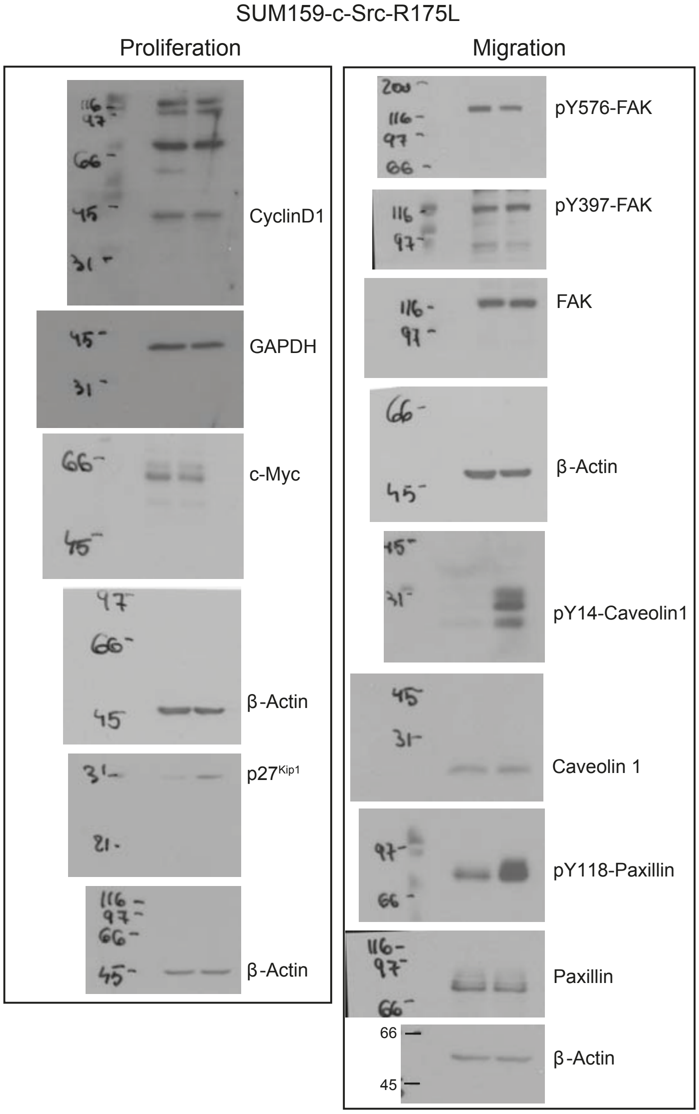

Uncropped gels corresponding to Figures 3, S7, 4, S9, S12 and S13.

Western Blots for Proliferation and Migration

SUM159-c-Src-R175L/W118A

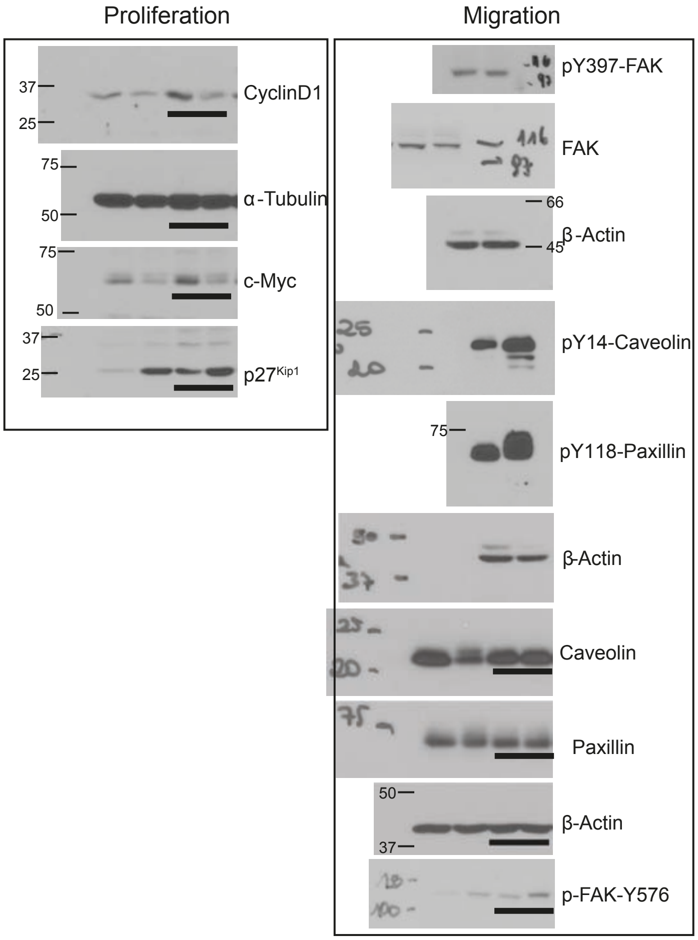

Uncropped gels corresponding to Figures 3, S7, 4, S9, S12 and S13.

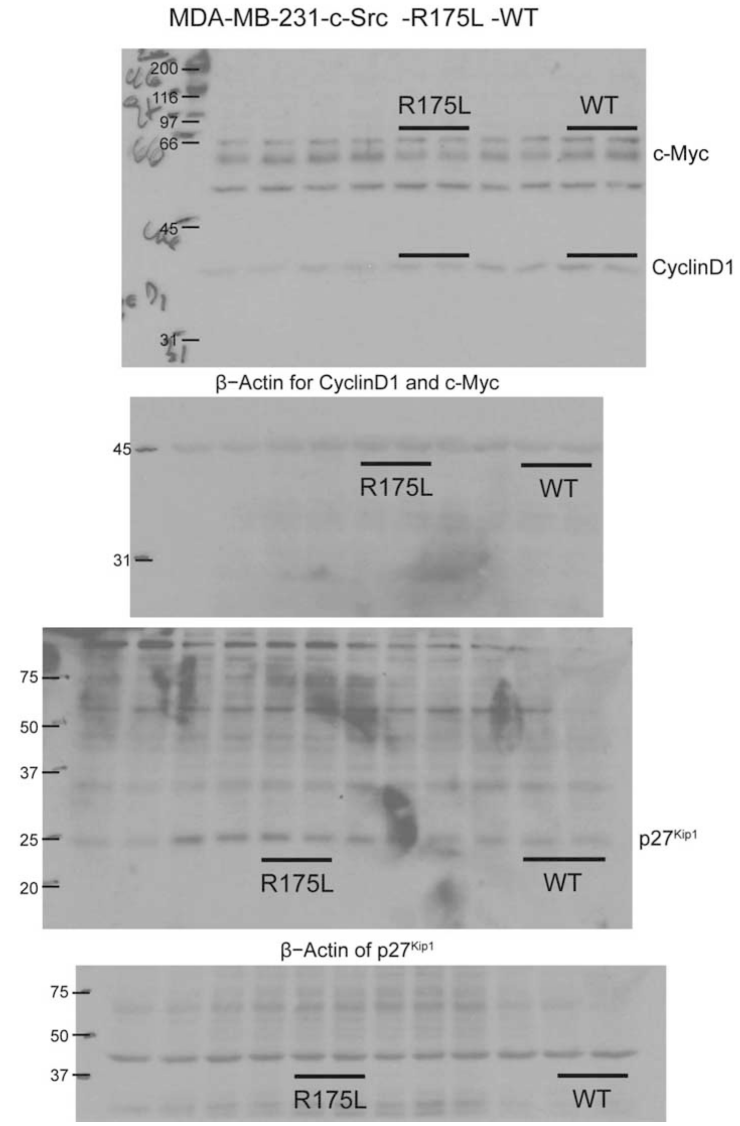

Uncropped gels corresponding to Uncropped gels corresponding to Figures 3, S7, 4, S9, S12 and S13.

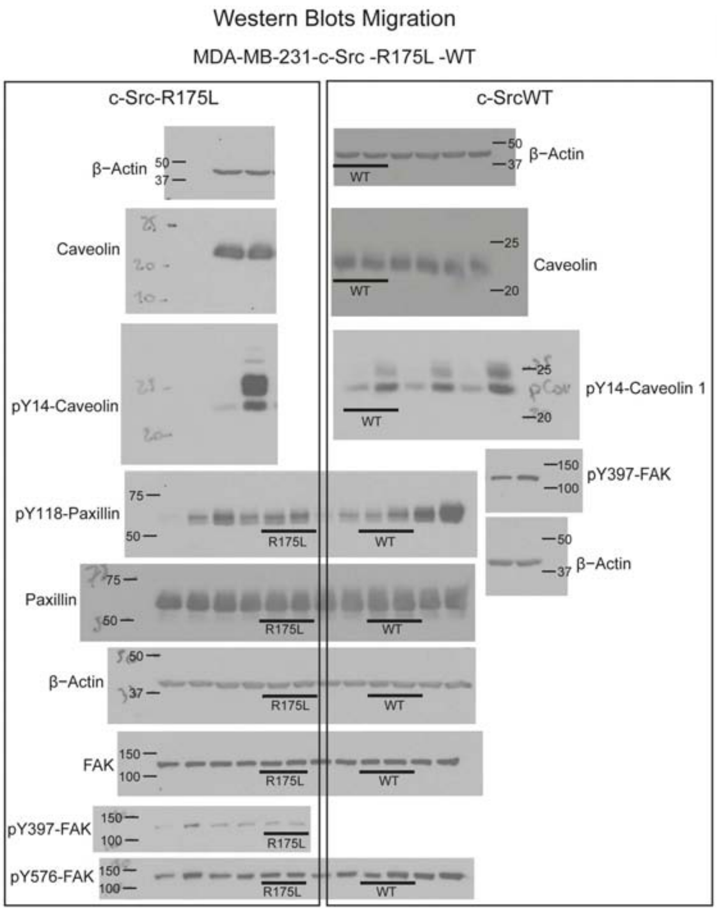

Uncropped gels corresponding to Figures 3, S7, 4, S9, S12 and S13.

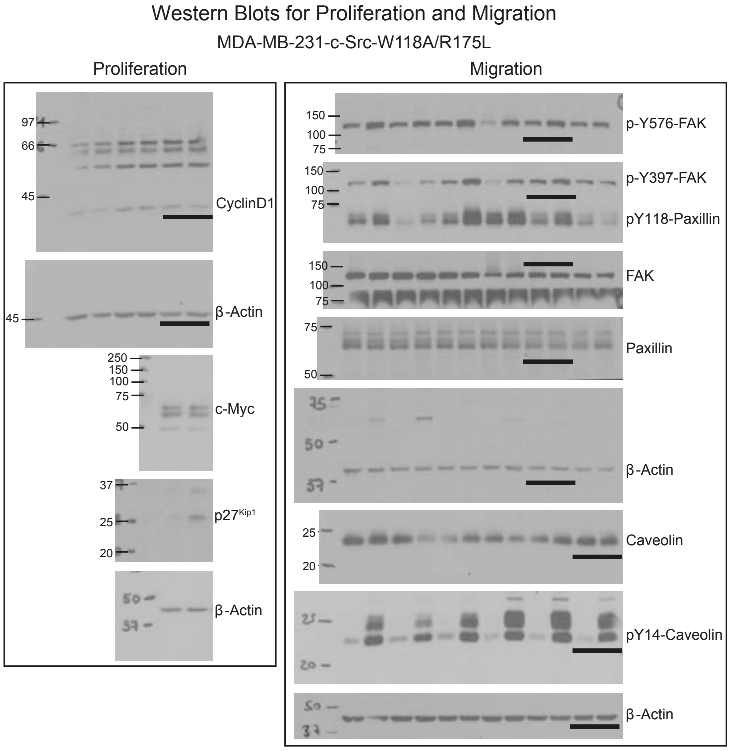

Uncropped gels corresponding to Figures 3, S7, 4, S9, S12 and S13.

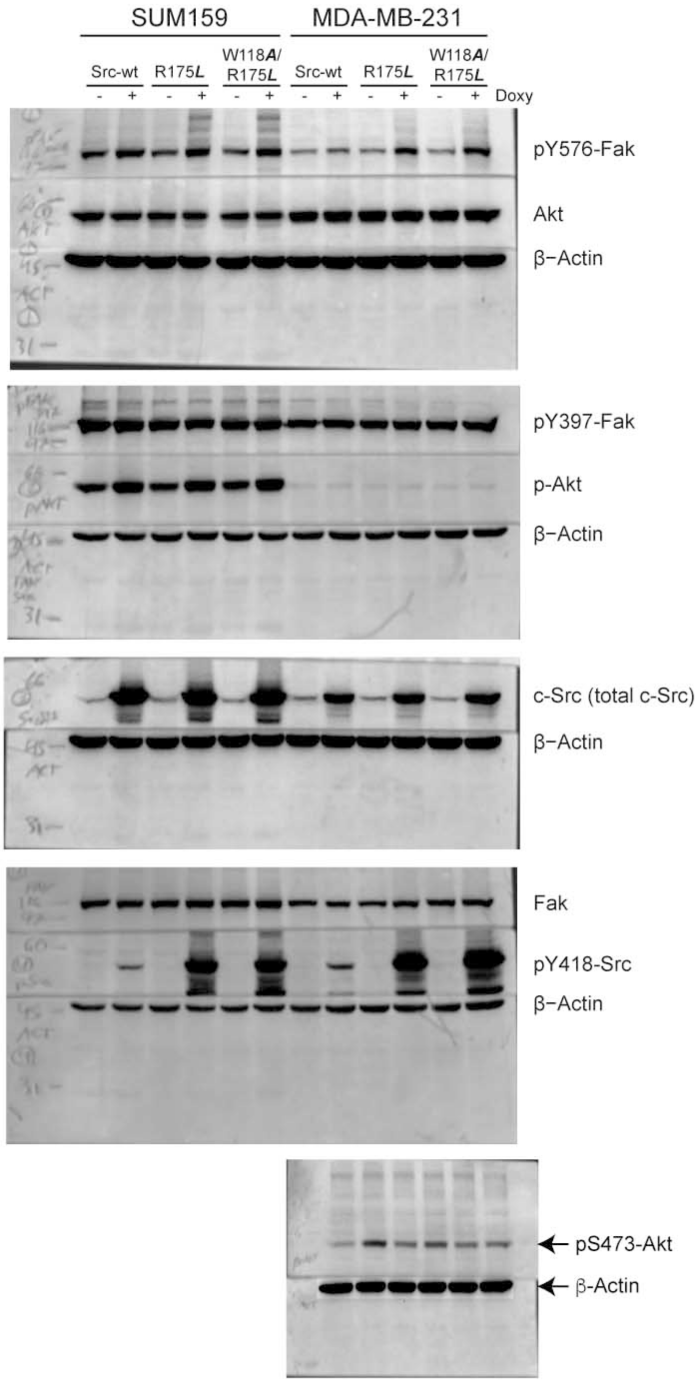

Uncropped gels corresponding to Supplementary Figure S14.
Since the intensity of the pS473-Akt signals between SUM157 and MDA-MB-231 cells was too different, we reblotted again the part of the membrane corresponding to MDA-MB-231 samples with antibodies to pS473-Akt and β-Actin, as a loading control.
